# Molecular Engineering of Adeno-Associated Virus Capsid Improves Its Therapeutic Gene Transfer in Murine Models of Hemophilia and Retinal Degeneration

**DOI:** 10.1021/acs.molpharmaceut.9b00959

**Published:** 2019-10-22

**Authors:** Bertin Mary, Shubham Maurya, Mohit Kumar, Sridhar Bammidi, Vikas Kumar, Giridhara R. Jayandharan

**Affiliations:** † Department of Biological Sciences and Bioengineering, Indian Institute of Technology Kanpur, Kanpur 208 016, Uttar Pradesh, India; ‡ Mass Spectrometry and Proteomics Core Facility, University of Nebraska Medical Center, Omaha 68198, Nebraska, United States

**Keywords:** AAV2, glycosylation, hemophilia B, Leber congenital amaurosis

## Abstract

Recombinant adeno-associated virus (AAV)-based gene therapy has been promising, but several host-related transduction or immune challenges remain. For this mode of therapy to be widely applicable, it is crucial to develop high transduction and permeating vectors that infect the target at significantly low doses. Because glycosylation of capsid proteins is known to be rate limiting in the life cycle of many viruses, we reasoned that perturbation of glycosylation sites in AAV2 capsid will enhance gene delivery. In our first set experiments, pharmacological modulation of the glycosylation status in host cells, modestly decreased (1-fold) AAV2 packaging efficacy while it improved their gene expression (~74%) in vitro. We then generated 24 mutant AAV2 vectors modified to potentially create or disrupt a glycosylation site in its capsid. Three of them demonstrated a 1.3–2.5-fold increase in transgene expression in multiple cell lines (HeLa, Huh7, and ARPE-19). Hepatic gene transfer of these vectors in hemophilia B mice, resulted in a 2-fold increase in human coagulation factor (F)IX levels, while its T/B-cell immunogenic response was unaltered. Subsequently, intravitreal gene transfer of glycosylation site-modified vectors in C57BL6/J mice demonstrated an increase in green fluorescence protein expression (~2- to 4-fold) and enhanced permeation across retina. Subretinal administration of these modified vectors containing RPE65 gene further rescued the photoreceptor response in a murine model of Leber congenital amarousis. Our studies highlight the translational potential of glycosylation site-modified AAV2 vectors for hepatic and ocular gene therapy applications.

## Introduction

Recombinant adeno-associated virus (AAV) vectors based on serotype 2 have gained prominence in gene therapy applications because of its excellent safety profile. During the last decade, AAV2 has been used successfully for in vivo gene transfer in several preclinical animal models with a long-term expression noted for a wide variety of therapeutic genes.^1,2^ In human clinical trials, after initial successful phenotypic correction in patients with hemophilia B or Leber congenital amaurosis type 2 (LCA2), a lack of substantial gene expression from AAV2 vectors^3^ or vector-related cellular immune response has been noted.^4,5^ Although other serotypes including AAV5 and AAV8 have been successfully employed for gene therapy of hemophilia B or LCA2,^6^ AAV2 is the widely studied serotype (>6.9% of gene therapy trials) and its unrestricted use in clinical space has been widely beneficial to investigators in the gene therapy field. Thus, strategies to improve transduction of AAV2 vectors in the liver or eye would be beneficial for this mode of therapy to be widely embraced.

AAV2 capsid protein, which constitutes 74% of the vector is the major source of foreign antigen during gene therapy,^7^ but is also an essential component for host cell receptor recognition, intracellular trafficking, and T-and B-cell recognition.^8^ Thus, a thorough study and modification of the capsid regions that trigger vector recognition and loss of function is likely to improve gene transfer. Previously, AAV2 vectors which can bypass intracellular phosphorylation and subsequent proteosomal capsid degradation had shown significantly higher transgene expression after hepatic or ocular gene transfer.^9^ Given the paucity of data on other post translational modifications (PTM) in AAV,^10,11^ we reasoned that a study of glycosylation motifs in the AAV2 capsid is likely to be rewarding, as its role in several viruses such as HIV and influenza is known.^12^ When AAV2 vectors are packaged in human cervical carcinoma (HeLa) cells, with the helper function provided by live adenovirus, glycosylation was not identified at a detection limit of 10% of VP1 protein.^10^ In contrast, Kaludov et al. reported that cellular glycosylation has an impact on the binding and transduction efficiency of AAV4 and AAV5 serotypes.^13^ More recently, a high throughput analysis identified that the AAV8 capsid is glycosylated at the N499 residue.^14^ Several AAV serotypes such as AAV1, 2, 6, and 9 utilize glycans as their cellular receptors and coreceptors.^15–17^ Recently, we have studied the role of different PTMs in multiple AAV capsids (AAV2-rh10 serotypes) in a packaging cell line by advanced mass spectrometric analysis.^18^ Taking into account these observations, we studied the role of cellular glycosylation in the AAV2 life cycle, by use of an expanded panel of glycosylation modulators and assessed their impact on transduction and packaging efficiency. Based on our initial findings, we reasoned that perturbation of putative T-cell and B-cell recognition epitopes and addition or deletion of glycosylation targets within these regions in the AAV2 capsid will lead to the generation of efficient AAV2 vectors.

## Experimental Section

### Cell Lines and Reagents

Human cervical carcinoma cell line (HeLa), hepatocellular carcinoma cell line (Huh7), and AAV packaging cell line (AAV293) was purchased from the American Type Culture Collection (ATCC, Rockville, MD, USA). Cells were maintained as monolayer cultures in Iscove’s modified Dulbecco’s medium (Gibco Life Technologies, Carlsbad, CA, USA) supplemented with 10% fetal bovine serum (Gibco), 10 *μ*g/mL piperacillin (MP Biomedicals, Illkirch, France), 10 *μ*g/mL ciprofloxacin (HiMedia Laboratories, Mumbai, India) and sodium bicarbonate (Sigma-Aldrich, St Louis, MO, USA). The cell lines used in this study were authenticated by short tandem repeat profiling (data not shown). Antibodies such as FITC-anti CD3, PerCP-anti CD4, PE-anti CD8, APC-anti CD19, PE-anti Foxp3, and APC-anti CD25 for immune assays were from BD Biosciences (Franklin Lakes, NJ, USA).

### Generation of Recombinant AAV Vectors

Recombinant self-complementary (sc)AAV2 vectors containing enhanced green fluorescence protein (EGFP) gene driven by the chicken *β*-actin promoter and bovine growth hormone poly (A) signal, human coagulation factor FIX (hFIX) gene driven by a LP1 promoter/enhancer, or single-stranded (ss)AAV2 human retinal pigmental epithelium gene encoding 65 kDa (hRPE65) under the control of the cytomegalovirus promoter were generated by the triple transfection protocol as described previously.^19^ Purified vectors were measured by a quantitative PCR (qPCR)-based assay using polyA region specific primers after DNase treatment (Sigma-Aldrich) as described previously.^20^ The vector titers are expressed as viral genomes (vg)/mL.

### Modulation of Cellular Glycosylation by Small Molecules

To assess the effect of modulation of cellular glycosylation on AAV2 transduction, cells (HeLa, Huh7, and ARPE-19) were mock [phosphate-buffered saline (PBS)]-treated or pretreated with an optimal concentration of small molecules for the specified duration (Table S1). Subsequently, these cells were infected with scAAV2-EGFP vectors at a multiplicity of infection (MOI) of 1 × 10^3^ vgs/cell. Forty-eight hours later, GFP expression was assessed by flow cytometry. Mean of percentage GFP positivity from two independent experiments were used for comparison between mock and AAV vector-infected cells in a BD Accuri C6 Plus flow cytometer (BD Biosciences, Franklin Lakes, NJ, USA).

### Generation of AAV Vectors in the Presence of Glycosylation Modulators

AAV2 vectors were generated in the packaging cell line, AAV293 in the presence or absence of pharmacological modulators of glycosylation. Two sets of packaging experiments were performed to understand the efficiency and dynamics of glycosylation modulation during the packaging process. In the first set of packaging experiments, we pretreated the AAV293 cells with glycosylation modulators prior to triple transfection with plasmid vectors. Briefly, scAAV2-EGFP vectors were packaged in mock treated cells or in AAV293 cells pretreated with a N-linked glycosylation inhibitor, tunicamycin (0.125 *μ*g/mL) or a glycosyltransferase enhancer, all-trans retinoic acid (ATRA, 1 *μ*M) for 24 and 48 h, respectively. In the next set of studies, the AAV293 cells were treated with glycosylation modulators 6 h after the initial step of transfection of plasmids. Briefly, 20 plates of transfected AAV-293 cells were treated with an O-linked glycosylation inhibitor, Alloxane (5 mM) for 12 h and another set of 20 transfected plates were treated with N-linked glycosylation inhibitor (tunicamycin) for 24 h. Twenty transfected plates were maintained as a mock control. Cells were harvested after 72 h and the vectors purified and quantified as described above.

### In Silico Analysis of the AAV2 Capsid Protein for Immune Epitope and Glycosite Modification

Antigenicity of the AAV2 capsid protein was predicted using B-cell and T-cell linear epitope mapping tools, IEDB resource server (http://tools.iedb.org/bcell/, http://tools.iedb.org/main/tcell/). We further refined the prediction results, with experimentally validated epitopes published previously,^21–24^ to define targets for glycosylation modifications using NetNGlyc (http://www.cbs.dtu.dk/services/NetNGlyc/) and MotifScan (https://myhits.isb-sib.ch/cgi-bin/motif_scan) tools. Similarly, potential O-glycosylation motifs were searched using NetOGlyc prediction servers (http://www.cbs.dtu.dk/services/NetOGlyc/). Thus, the refined epitopes were targets for mutagenesis by introducing or abolishing N- and O-linked glycosylation motifs in the AAV2 capsid.

### Generation of Glycosylation Site-Modified AAV2 Vectors

Site-directed mutagenesis of the target amino acids (N→Q, Q→N, S→A, A→T, E/K/Q/R→T, and Y/S/T→N) in AAV2 capsid was performed using a QuikChange II site-directed mutagenesis kit (Agilent Technologies, Santa Clara, CA, USA) as per the manufacturer’s protocol using specific primers (Table S2). The presence of mutations was confirmed by DNA sequencing. Viral vectors with glycosylation site-modified capsids were packaged, purified and titers measured by a qPCR protocol as described above.

### Transduction Assays with Glycosylation Site-Modified AAV2 Vectors

To assess the efficacy of the AAV2 glycosylation site-modified vectors, an infectivity assay was performed in three different cell lines, namely, HeLa, Huh7, and ARPE-19. Approximately, 3 × 10^4^ cells in culture were infected with 5 × 10^3^ vgs/cell of scAAV2-EGFP (wildtype, WT) or AAV2 glycosylation site-modified mutant vectors. Forty-eight hours post-infection, GFP expression was quantified by flow cytometry (BD Accuri C6 Plus). Data from two independent biological experiments with three technical replicates in each experiment were included in the analysis.

### Neutralization Assays

To assess the ability of glycomutants to escape neutralizing antibodies (Nab), we performed a neutralization assay with intravenous immunoglobulin (IVIG) as described previously.^25^ Briefly, AAV2-WT vectors (~5 × 10^3^ vgs) were incubated with two-fold dilution (1:4 to 1:2048) of IVIG (Kiovig, Baxter, Lessines, Belgium) at room temperature for an hour. After stimulation with etoposide (10 *μ*M) (Sigma-Aldrich, USA), AAV2-WT vectors either alone or in combination with different concentrations of IVIG were used to infect HeLa cells. Two days later, the transgene (EGFP) expression was measured by flow cytometry (BD Accuri C6 Plus). The highest dilution titer of IVIG in which the number of GFP positive cells was 50% lower than IVIG-free AAV2 control was considered as the Nab titer of the AAV2 vector. Once the Nab titer of IVIG for AAV2-WT was established, the AAV2 glycosylation site-modified mutants were screened at this predefined IVIG concentration, for their ability to escape the Nab.

### Estimation of Human Coagulation FIX Transcripts by qPCR

Huh7 cells were infected with either AAV2-WT or glycosylation site modified vectors expressing hFIX at a MOI of 5 × 10^4^ vgs. Forty-eight hours later, total RNA was isolated by TRIzol reagent (Ambion, Life technologies, Carlsbad, USA) and a qPCR for hFIX expression was done in a CFX96 real-time system (Biorad, Hercules, California, USA) using the primers detailed in Table S3. Normalization of data was performed with *β*-actin and the relative levels of FIX gene expression were calculated against AAV2-WT transduced cells by comparative Ct method (ΔΔCt).^26^ Data from two independent biological experiments with three technical replicates were included in the analysis.

### Characterization of Glycosylation in the AAV2-T14N Vector by Mass-Spectrometry

About 100 *μ*g of protein extract from AAV2 vectors was re-suspended in 100 mM ammonium bicarbonate and digested with MS-grade trypsin/Lys-C (Pierce, Thermo Fisher Scientific, Waltham, MA, USA) overnight at 37 °C. Peptides were cleaned with PepClean C18 spin columns (Thermo Fisher). Samples were re-suspended in 2% acetonitrile (ACN) and 0.1% formic acid (FA) and loaded onto trap column [Acclaim PepMap 100 75 *μ*m × 2 cm C18 LC Columns (Thermo Fisher)] at a flow rate of 4 *μ*L/min. Samples were then separated in a Thermo RSLC Ultimate 3000 on a Thermo Easy-Spray PepMap RSLC (75 *μ*m × 50 cm) 2 *μ*m C-18 column (Thermo Fisher) with a step gradient of 4–25% solvent B (0.1% FA in 80% ACN) from 10 to 57 min and 25–45% solvent B for 57–62 min at 300 nL/min and 50 °C, with a 90 min total run time. Eluted peptides were analyzed by a Thermo Orbitrap Fusion Lumos Tribrid (Thermo Fisher) mass spectrometer in a data-dependent acquisition mode.

A survey full scan MS (from *m*/*z* 350 to 1800) was acquired in the Orbitrap with a resolution of 120 000. The AGC target for MS1 was set as 4 × 10^5^ and ion filling time set as 100 ms. The most intense ions with charge state 2–6 were isolated in 3 s cycle and fragmented using HCD fragmentation with 40% normalized collision energy and detected at a mass resolution of 30 000 at 200 *m*/*z*. The AGC target for MS/MS was set as 5 × 10^4^ and ion-filling time set 60 ms dynamic exclusion was set for 30 s with a 10 ppm mass window. Protein identification was performed by searching MS/MS data against the AAV sequence database. The search was set up for full tryptic peptides with a maximum of two missed cleavage sites. Acetylation of protein N-terminus, oxidized methionine, HexNAc of asparagine, phosphorylation, ubiquitination, and SUMOylation were included as variable modifications and carbamidomethylation of cysteine was set as fixed modification. The precursor mass tolerance threshold was set to 10 ppm and maximum fragment mass error was 0.02 Da. Qualitative analysis was performed using PEAKS X software (Bioinformatics Solutions Inc., Waterloo, ON, Canada).

### Animal Procedures

All animal studies were performed after approval by the Institute Animal Ethics Committee at the Indian Institute of Technology, Kanpur, India. Hepatic gene transfer studies involved the use of hemophilia B (B6.129P2-*F9^tm1Dws^*/J) mice, and ocular gene transfer was performed in C57BL6/J mice or in the rd12 (Leber congenital amarousis model, B6(A)-*Rpe65^rd12^*/J). Hemophilia B, rd12, and C57BL6/J mice were purchased from Jackson Laboratory (Bar Harbor, ME, USA).

### Hepatic Gene Transfer in Hemophilia B Mice

Hemophilia B mice (*n* = 5/group) were injected intravenously with either PBS (mock) or wild-type or glycosylation site-modified AAV2 vectors expressing hFIX at a dose of 5 × 10^10^ vgs per animal. Blood samples in 3.8% citrate buffer was collected from the retro-orbital plexus at 4, 10, and 12 weeks after gene transfer, to measure plasma FIX antigen levels by the enzyme-linked immunosorbent assay (ELISA) as per the manufacturer’s protocol (Asserachrom FIX: Antigen Kit, Diagnostica Stago, France).

### T-Cell and B-Cell Assays after FIX Hepatic Gene Transfer

To examine the immunogenicity of the AAV2-hFIX gene delivery protocol, we assessed the T-cell, B-cell, and regulatory T-cell (Treg) population in experimental animals, 12 weeks after gene transfer. After red blood cell lysis, pelleted cells were incubated with FITC-labeled anti-CD3, PE-labeled anti-CD8, PerCP-labeled anti-CD4, and APC-labeled anti-CD19 antibodies for 30 min at room temperature. The percentage CD3+, CD4+, CD8+, and CD19+ cells were then assessed by flow cytometry (BD Accuri C6 Plus). These data were used to enumerate B-cells (CD19+) and the double positive markers among the CD3+ population including, CD4 helper cells (CD3+ CD4+), CD8 cytotoxic cells (CD3+CD8+) in each of the AAV2 vector or PBS-administered mice (Figure S1).

To estimate the percentage Treg population in mouse splenocytes, ~1 × 10^6^ cells were stained with PerCP-labeled anti-CD4 and APC-labeled anti-CD25 antibodies for 30 min. Subsequently, cells were washed, fixed, and permeabilized using the mouse Foxp3 buffer set (BD Pharminogen) and further stained with the PE-conjugated Foxp3 antibody for 30 min. Flow cytometry was performed to enumerate the Treg (CD4+ CD25+ Foxp3+) cells.

### ELISPOT Assay

To measure CD8+ T cell-specific immune response, hemophilia B mice (*N* = 5/group) were injected with PBS (mock), AAV2-WT, or AAV2-T14N expressing hFIX at a dose of 5 × 10^10^ vgs. Mouse splenocytes were isolated from the spleen of treated and control animals, 9 weeks post gene transfer. After RBC lysis, ~1 × 10^6^ cells were seeded per well in a 96 well IFN-*γ* antibody precoated ELISPOT plate (MabTech, Cincinnati, OH, USA). Cells were then stimulated with 2 *μ*g/mL of AAV2 serotype, T-cell epitope-specific peptide (SNYNKSVNV) (JPT Peptide Technologies, GmbH, Germany) overnight and the ELISPOT assay was performed as per the manufacturer’s protocol. Concanavalin A (2 *μ*g/mL) was used as the positive control for the assay. Plates were incubated for 24 h and developed using BCIP/NBT. Spot-forming units and the images of the wells were captured in an ELISPOT reader (AID reader, GmbH, Germany).

### Intravitreal Gene Transfer with AAV-GFP Vectors and Fluorescence Imaging in Vivo

Eyes of C57BL6/J mice were dilated by 1% atropine (Jawa Pharmaceuticals India Pvt. Ltd, Gurgaon, India), phenylephrine + tropicamide (Sunways India Pvt. Ltd. Mumbai, India). The mice were anesthetized by intraperitoneal injection of ketamine (80 mg/kg) and xylazine (12 mg/kg). For intravitreal administration, an opening was created at the sclera near the limbus by an insulin syringe, and 1 *μ*L of AAV vector (3 × 10^8^ vgs) was injected through the same opening by a Hamilton syringe containing a a 33-gauge beveled needle. Fluorescence imaging was performed after 4 and 6 weeks of vector administration in a Micron IV imaging system (Phoenix Research Lab, Pleasanton, CA, USA).

### Immunohistochemical Analysis

For immunostaining of liver sections for FIX expression, murine liver was embedded in OCT media (Polyfreeze, Sigma-Aldrich) and after sectioning was fixed with 4% paraformaldehyde. Tissue sections were blocked with 10% normal donkey serum (Santa Cruz Biotechnology, Dallas, TX, USA) and then incubated with goat antihuman FIX (Affinity Biologicals, Hamilton, ON, Canada) overnight at 4 °C. Samples were probed with the donkey antigoat Cy3 antibody (Jackson ImmunoResearch, West Grove, PA, USA). For nuclear staining, 4,6-diamidino-2-phenylindole was used. Images were acquired in a Leica DMi8 confocal microscope (Wetzlar, Germany).

For immunostaining of retinal sections, eye balls from the control and AAV2-treated mice were harvested after 6 weeks of gene transfer.^27^ Cryosectioned retinal sections were permeabilized with 0.5% Triton X-100 for 15 min, followed by incubation with the blocking agent (10% normal goat serum, Abcam, Cambridge, UK) for 1 h. The retinal sections were then incubated with a mouse monoclonal antibody to GFP at a 1:50 dilution (Abcam) in 10% normal goat serum and further stained by the FITC-labeled rabbit antimouse antibody (Abcam, Cambridge, UK). For nuclear staining, we used 4,6-diamidino-2-phenylindole. The retinal sections were imaged by confocal microscopy (Leica DMi8).

### Intravitreal Administration of AAV2 Vectors Packaged in the Presence of Tunicamycin or Treated with Glycosidase Enzyme

AAV2 glycosylation site-modified (T14N) vectors packaged conventionally or under a cellular glycosylation inhibited condition, in the presence of tunicamycin was administered intravitreally at a dose of 2 × 10^8^ vgs into C57BL6/J mice (*n* = 4 eyes per group). Two weeks later, the retinal sections (*n* = 3 sections per eye) were imaged by confocal microscopy (LSM780NLO, Carl Zeiss, Oberkochen, Germany) to assess transgene (EGFP) expression and the permeation of the vector across the neural retina.

Similarly, AAV2-T14N vectors expressing EGFP, at a dose of 3 × 10^8^ vgs was treated with 250U of a glycosidase enzyme (PNGase F, New England Biolabs, Ipswich, MA, USA) overnight at 37 °C. After treatment, the vectors were administered into C57BL6/J murine eyes by intravitreal injection (*n* = 5 eyes/group). Four weeks after gene transfer, murine eyes were eunucleated and retinal sections (*n* = 3 sections per eye) were prepared. Confocal imaging was performed to assess the transduction and permeation of the vectors after treatment with the glycosidase. Animals administered with only AAV2-T14N vectors were included as experimental controls.

### Subretinal Administration of AAV-RPE65 Vectors and Electroretinography

Approximately, 1 × 10^8^ vgs or 7 × 10^8^ vgs/eye of wild-type or glycosylation site-modified AAV2 vector (AAV2-Q259N or AAV2-T14N, respectively) expressing hRPE65 were administered via the subretinal route into groups of 8 week-old rd12 mice. Subretinal injections were carried out by the following procedure. The corneal–scleral junction at the limbus was pricked with a beveled 31G needle, releasing the pressure. Care was taken not to injure the cornea and lens. A 33G blunt needle attached to a Hamilton microsyringe was introduced through the aperture at the cornea–scleral junction and it was taken across the vitreous to the retina. The retina/RPE junction was reached by analyzing the back pressure and the volume injected in a jet into the space without exerting pressure on the needle or the RPE. Fundus images were acquired post-surgery to check if there was any detachment. A detached bleb, signified successful subretinal administration.

Scotopic electroretinography (ERG) was further carried out 6, 10, or 32 weeks later to study the visual physiology. Mice were dark-adapted overnight to completely flush any residual phototransduction activity. Animals were anaesthetized and the pupil was dilated with tropicamide/phenylephrine solution. Further, they were placed on a heating pad, to prevent hypothermia, and electrodes viz. corneal contact (objective lens) tail (ground) and head (reference) of the Ganzfeld ERG system (Phoenix Research Labs, Pleasanton, CA, USA), were placed, respectively. The cornea was moistened with 2% hypromellose topical solution and the positive electrode was placed on the cornea. Eyes were illuminated with flashes of light stimuli (−1.7 to 3.1 log cd s/m^2^) and electrical responses were recorded and analyzed using Labscribe software (Labscribe, iWorx systems, Dover, NH, USA). Representative ERG wave forms were acquired for qualitative analysis and the mean of the amplitudes was plotted against a-wave and b-wave.

### Statistical Analysis

Comparisons between the test and control groups were performed with GraphPad Prism software (GraphPad, La Jolla, CA, USA) by Student’s *t*-test or by ANOVA. A *p*-value < 0.05 was considered to be statistically significant.

## Results

### Effect of Glycosylation Modulators on AAV-Mediated Gene Expression in Vitro

**Figure 1:**
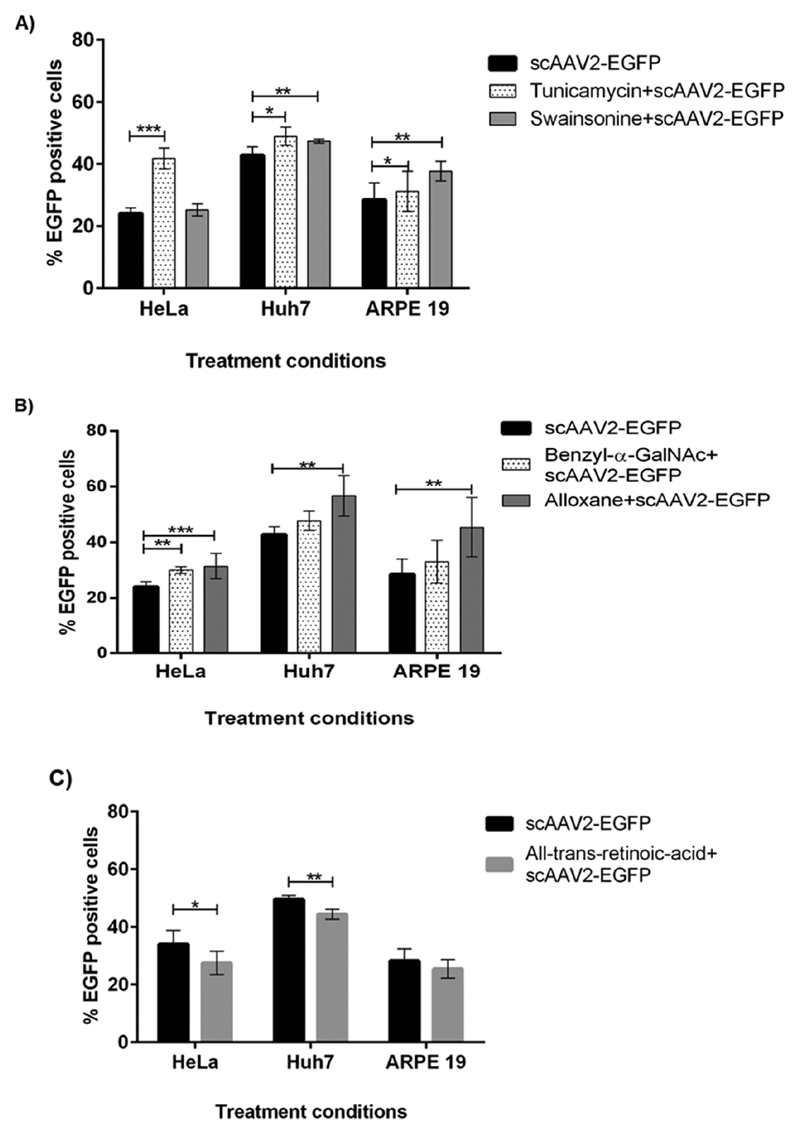
In vitro transduction efficiency of AAV2 vectors in HeLa, Huh7, and ARPE-19 cells in the presence of glycosylation modulators. (A) Cells were pretreated with N-glycosylation inhibitors—tunicamycin and swainsonine or (B) O-glycosylation inhibitors—benzyl-α-GalNAc and alloxane (C) glycosylation enhancer—all-trans retinoic acid and infected with scAAV2-EGFP at 1 × 10^3^ vgs/cell. The transduction efficiency of these conditions was compared to scAAV2 alone treated cells. *N* = 6 replicates, *p < 0.05, **p < 0.01, ***p < 0.001 vs scAAV2-WT-treated cells.

To check the effect of glycosylation inhibitors on AAV2 transduction, HeLa cells were cultured in the presence of N-linked and O-linked glycosylation inhibitors and further infected with scAAV2-EGFP vectors. We observed that pretreatment of HeLa cells with tunicamycin, an inhibitor of N-linked glycosylation, prior to AAV infection, enhanced GFP expression by >50% when compared to the mock infected cells, but only a modest increase (~5%) was seen with another N-linked glycosylation inhibitor, swainsonine. Similarly, pretreatment of HeLa cells with an O-linked glycosylation inhibitor, benzyl-*α*-GalNAc and alloxane increased the transduction of AAV2 vectors by ~25% and ~30%, respectively (Figure 1A,B). This increase in AAV2 transduction may be because of the activity of inhibitors on glycosylation-specific cellular proteins, which are known to play a role in intracellular trafficking as reported for other viruses such as the Hantaan virus.^28^ To examine if an increase in AAV2 transduction upon inhibition of glycosylation status is cell-line specific, since cellular glycan expression pattern is hypervariable across cell lines,^29^ we performed similar experiments in Huh7 and ARPE-19 cells. In Huh-7 cells, pretreatment of cells with the O-linked inhibitor—alloxane showed a modest improvement in the transduction efficiency of scAAV2-EGFP vectors (~32%) (Figure 1A,B). It is known that the basal level of Huh7 cell glycosylation is altered and it can affect the cell growth, cell migration, and receptor binding,^29^ and thus the modest increase seen in this experiment could be attributed to this phenomenon. Pretreatment of ARPE-19 cells with N-linked inhibitors had a modest increase (~9% and ~30%) in transduction (Figure 1A,B). In the case of O-linked glycosylation inhibitors, pretreatment of ARPE-19 cells with alloxane also demonstrated an increased transduction (~60%) with AAV vectors (Figure 1A,B). These studies suggest that the inhibition of N-linked or O-linked glycosylation consistently improved AAV2 transduction in multiple cell lines.

We next wished to assess if the activation of a terminal glycosyltransferase in N-linked glycosylation modulates gene expression from AAV2 vectors. We thus utilized ATRA, an activator of glycosyltransferase at a concentration of 1 *μ*M, prior to the transduction of HeLa, Huh7, and ARPE-19 cells with scAAV2-EGFP vectors. We observed that the transduction efficiency of AAV2 was inhibited by 10–20% in ATRA-treated cells (Figure 1C). Taken together, our data suggest that inhibition of cellular glycosylation has a positive impact on AAV2 transduction.

### Recombinant AAV2 Packaging in the Presence of N-Linked Glycosylation Inhibitor and Activator

In order to evaluate the role of cellular glycosylation during the AAV packaging process, we generated AAV2 vectors expressing EGFP in the presence of pharmacological modulators of glycosylation. Briefly, AAV293 cells were mock-treated or pretreated with either a N-linked glycosylation inhibitor (tunicamycin, 0.125 *μ*g/mL) or N-linked glycosylation activator (ATRA, 1 *μ*M) followed by triple transfection with AAV2 packaging plasmids. The recombinant vectors thus generated were further quantified by qPCR. Our data demonstrate that the titer of vectors packaged under normal conditions was 2.02 × 10^11^ vgs/mL [median: 2.83 × 10^11^ vgs/mL; range: 1.91 × 10^11^ to 4.44 × 10^11^] while vectors packaged in producer cells treated with tunicamycin had substantially lower titers 6.82 × 10^9^ vgs/mL [median: 5.34 × 10^9^; range: 3.47 × 10^8^ to 1.46 × 10^10^] (*p* value = 0.04). Producer cells treated with ATRA also generated lower titers of vectors, ~1.51 × 1010 vgs/mL [median: 1.23 × 10^10^; range: 7.59 × 10^10^ to 2.63 × 10^10^] (*p* value = 0.02), as represented in Figure S2.

We further performed independent packaging experiments where the AAV293 cells were posttreated (after 6 h of transfection with helper plasmids) with glycosylation modulators. As shown in Figure S2, when tunicamycin and alloxane are added after the plasmid transfection, a decrease in the vector yield was noted. This suggests that any perturbation in the host cellular glycosylation pathway is likely to affect the vector capsid protein synthesis, its modification and maturation thus possibly leading to a lower vector yield.

### In Silico Analysis of AAV2 Capsid Protein to Identify Target Sites for Modification

Potential N-linked and O-linked glycosylation motifs in AAV2 capsid were identified by NetNGlyc, NetOGlyc, and MotifScan (Figure S3). Immune epitope regions of the VP1 capsid protein were predicted by the IEDB server and analyzed in comparison to experimentally validated targets in the literature. Putative residues which are located within or near the immune epitope region and are part of predicted glycosylation sites were considered for glycosylation site abolition (O^−^/N^−^ mutant). Residues which were part of the predicted epitope and capsid-loop region and that were amenable to host glycosylation motifs were considered for glycosylation introduction (O^+^/N^+^ mutant). The residues thus shortlisted for targeted mutagenesis have been mapped onto the VP1 capsid protein sequence of AAV2 (Figure S4). Selected residues were further analyzed by NetNGlyc and NetOGlyc tools to confirm their glycosylation potential (Table S4).

### Glycosylation Site-Modified Vectors Demonstrate Improved Transduction in Ocular and Hepatic Cells in Vitro

As can be seen in Table S5, out of the 24 mutant vectors generated, four (A204T, K314T, Y377N, and N335Q) had a consistently lower (by 2-log) packaging titers compared to scAAV2-WT vectors. Because an adequate dose of vectors were not available for in vivo studies, they were not tested further.

**Figure 2:**
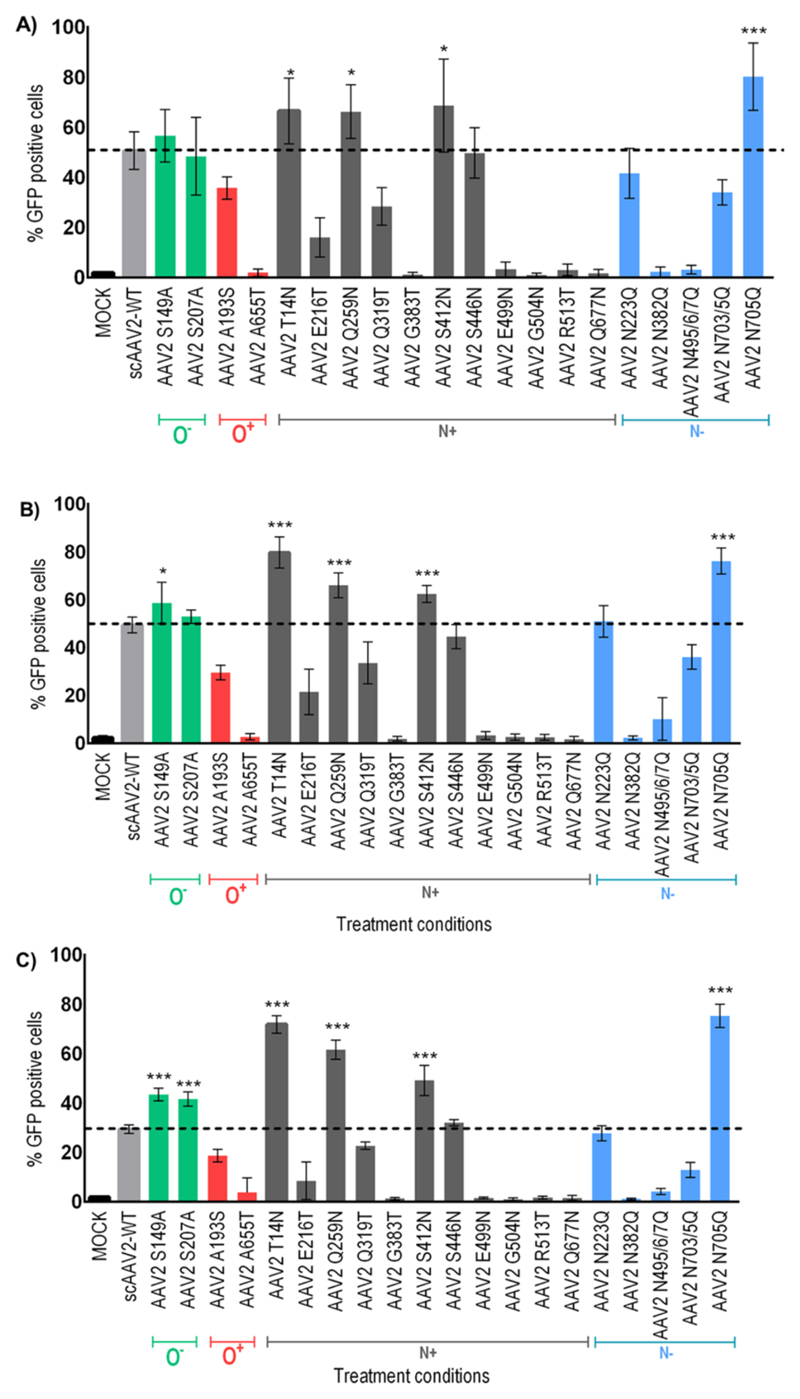
Transduction efficiency of AAV2 glycosylation site-modified vectors in human cells in vitro. HeLa cells were either mock-infected or infected with 5 × 10^3^ vgs/cell of AAV2-WT or AAV2 mutants (A) and cells analyzed for EGFP expression 48 h later by flow cytometry. The percentage EGFP positive cells post-transduction are shown. Similar experiments were carried out in Huh7 (B) or ARPE-19 cells (C). The data depicted are the mean of two independent experiments (*n* = 6). *p < 0.05, ***p < 0.001 vs AAV2-WT infected cells.

To assess the efficacy of the remaining 20 mutant vectors, an infectivity assay was performed in three different cell lines, namely, HeLa, Huh7, and ARPE-19. A mutant vector T14N in which a N-linked glycosylation site was created (N^+^) demonstrated an average 25% increase across all cell lines. Similarly, few other N^+^ mutant vectors [Q259N (66%), S412N (68%)] or N^−^ vectors such as N705Q (80%) also had a higher transgene expression in comparison to cells infected with AAV2-WT vectors (51%) (Figure 2A). In the case of both hepatic or ocular cells (Figure 2B,C), AAV2 mutants T14N, Q259N, and N705Q showed a relatively higher transgene expression (>2 fold in ARPE-19 and >1.2-fold in Huh7) and thus chosen as candidates for further in vivo studies.

### Evaluation of the Glycosylation Site-Modified Mutants for Their Immune Escape Activity

**Figure 3:**
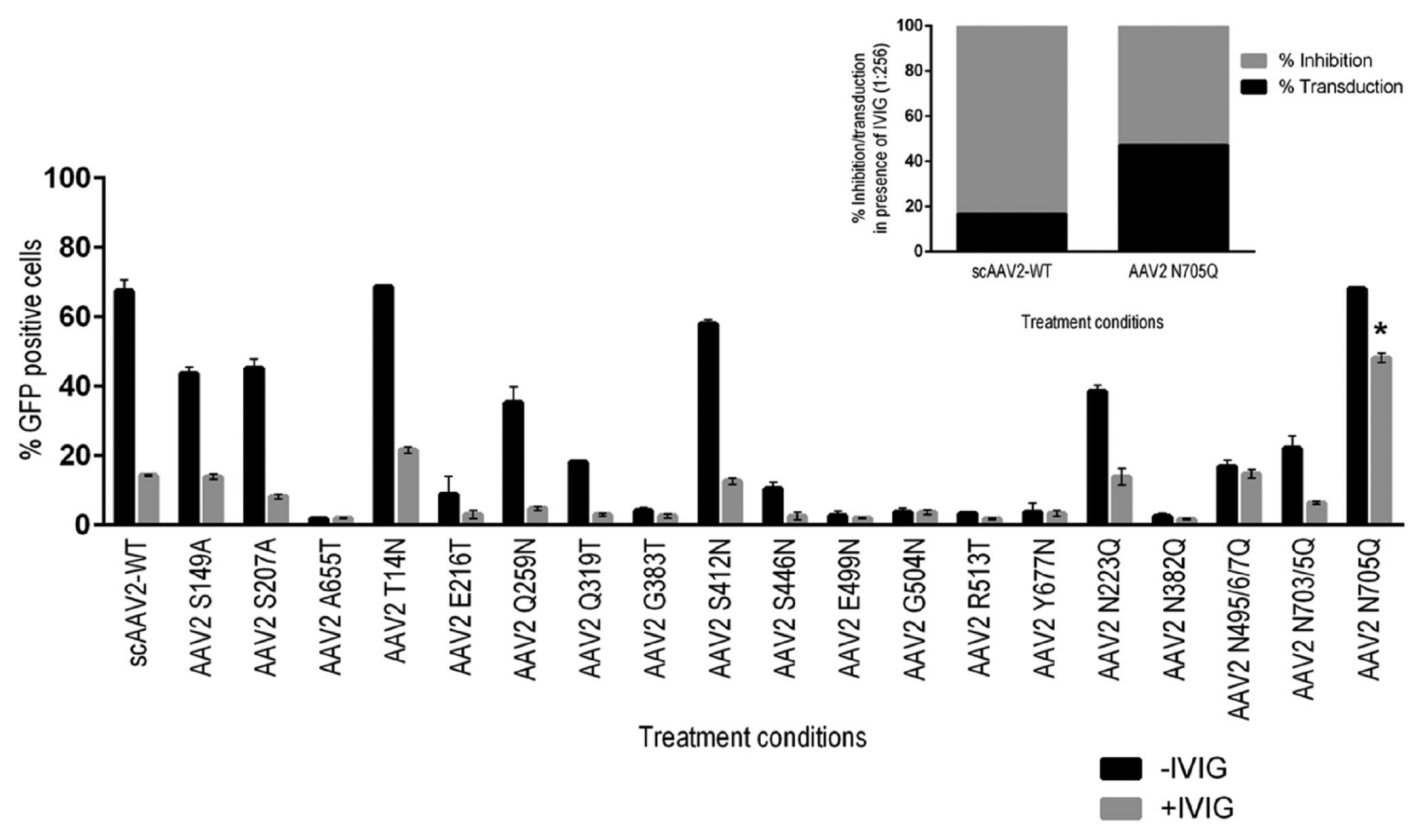
In vitro neutralization assay with AAV2 mutants in the presence or absence of IVIG. scAAV2-WT or mutant vectors were pre-incubated with IVIG at a dilution of 1:256 and assessed for their transduction in HeLa cells, 48 h later. The data is the representation of one independent experiment with three replicates. Inner subset image depicts the percent inhibition of transduction by scAAV2-WT and AAV2 N705Q vectors in the presence of IVIG. Data depicted in this panel is a representation of four independent experiments (*n* = 12). *p < 0.05 vs AAV2-WT infected cells without pretreatment with IVIG.

In our initial set of studies, we identified 1:256 dilution of IVIG as the Nab titer of the AAV2-WT vector (Figure S5). Based on this finding, we further evaluated other glycosylation site mutants at a IVIG titer of 1:256 to analyze their immune escape function. Of the 20 glycomutants, mutant A193S was not included in these assays because of their lower transduction (Figure 2). The N^−^ mutant, N705Q had a partial (~30%) immune escape function as measured by the increased transduction efficiency in the presence of IVIG [47 ± 15% vs 17 ± 3% in AAV2-WT-treated cells] (Figure 3).

### AAV2 T14N Vector Demonstrates Significantly Higher FIX Expression in Vitro and in Vivo

Based on our finding that AAV2 T14N, Q259N, and N705Q vectors had an enhanced GFP expression in the hepatic cell line Huh7, we further examined if these vectors could deliver a therapeutic gene such as FIX into hepatic cells in vitro. As can be seen inFigure S6, AAV2 T14N and N705Q-LP1 hFIX vectors exhibited a ~2.8-and 2.6-fold higher FIX mRNA expression.

**Figure 4:**
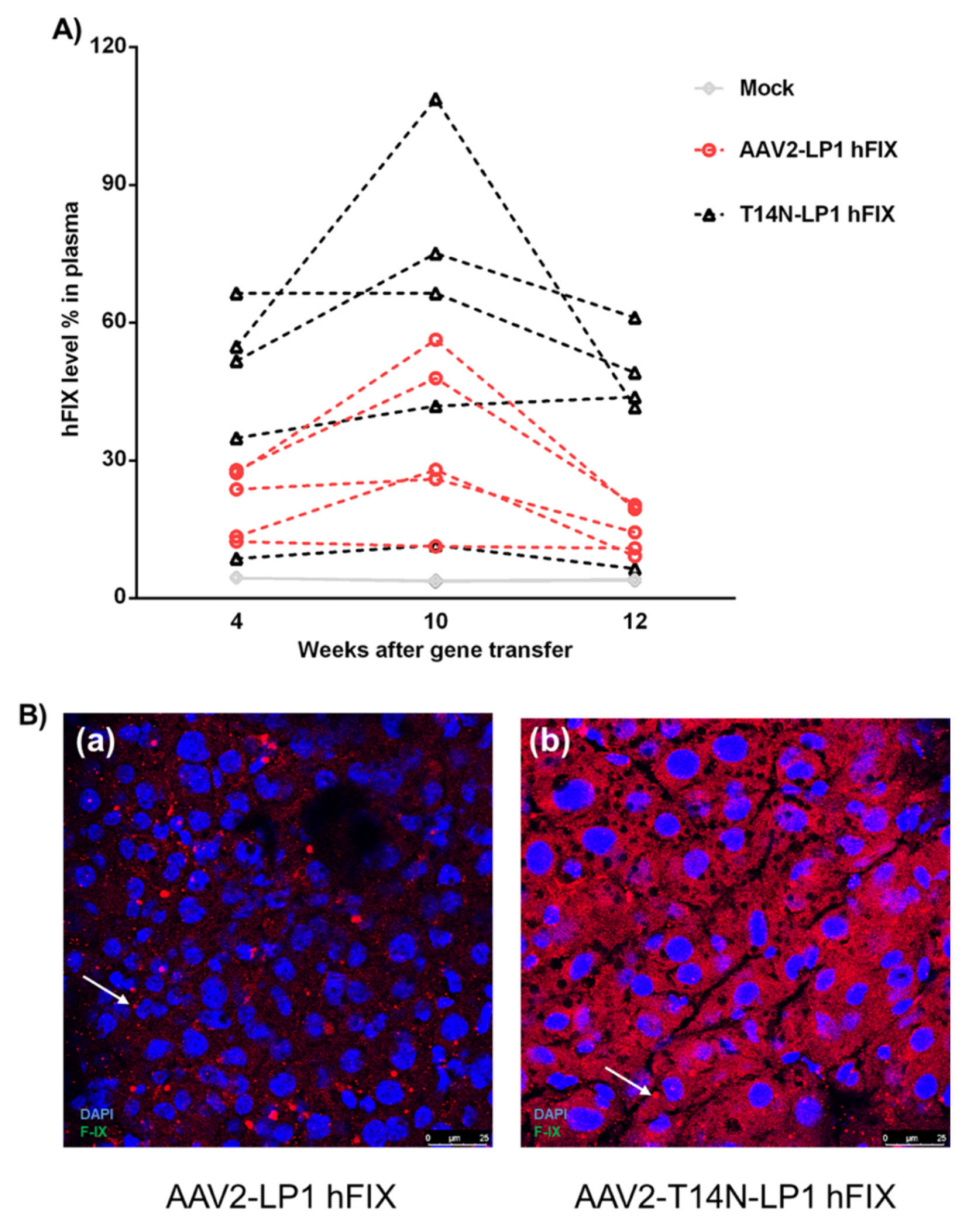
Glycosylation site-modified AAV2 vector demonstrate improved hepatic gene transfer of factor (F)IX in a murine model of hemophilia B. Groups of hemophilia B mice were administered with either PBS (mock, *n* = 5) or scAAV2-LP1-hFIX (*n* = 5) or scAAV2-T14N-LP1-hFIX (*n* = 5) vectors at a dose of 5 × 10^10^ vgs via tail vein. (A) Plasma levels of human FIX were measured at 4, 10, and 12 weeks after hepatic gene therapy. *p < 0.05 between AAV2-WT vs AAV2-T14N-injected mice at 4 and 12 weeks. (B) Representative images of immunohistochemistry for human FIX expression in scAAV2-LP1 hFIX and scAAV2-T14N-LP1-hFIX administered hemophilia B mice. Arrow marks denote the nonspecific signal seen in the immunostained section. Magnification is 400×.

Because T14N vectors demonstrated a sustained increase in EGFP or FIX expression in the hepatic cell line (Figures 2B and S6), we wished to further evaluate its potential for phenotypic correction in a murine model of hemophilia B. AAV2-WT or T14N-LP1 hFIX vectors were administered in groups of hemophilia B mice at a dose of 5 × 10^10^ vgs/animal, intravenously. Circulating hFIX levels were then estimated after 4, 10, and 12 weeks of hepatic gene transfer by ELISA (Figure 4A). The mean hFIX level in AAV2-FIX injected animals at 4, 10, and 12 weeks were 21 ± 7, 34 ± 18, and 14 ± 5% while hFIX levels in AAV2-T14N-LP1 hFIX-injected animals were 43 ± 22, 60 ± 36, and 40 ± 20%, respectively. This shows an average 1.8–2.7-fold increase for T14N mutants over the WT-AAV2 vectors. Further immunostaining done on liver sections from T14N- and WT-AAV2-treated animals showed an increase in FIX expression in T14N mutant administered mice (Figure 4B). Taken together, our data confirm the superior therapeutic efficiency of AAV2-T14N glycosylation site-modified vectors for hepatic gene transfer of diseases such as hemophilia B.

### T-Cell and B-Cell Response to AAV2-T14N Mutant is Unaltered after Hepatic Gene Transfer

**Figure 5:**
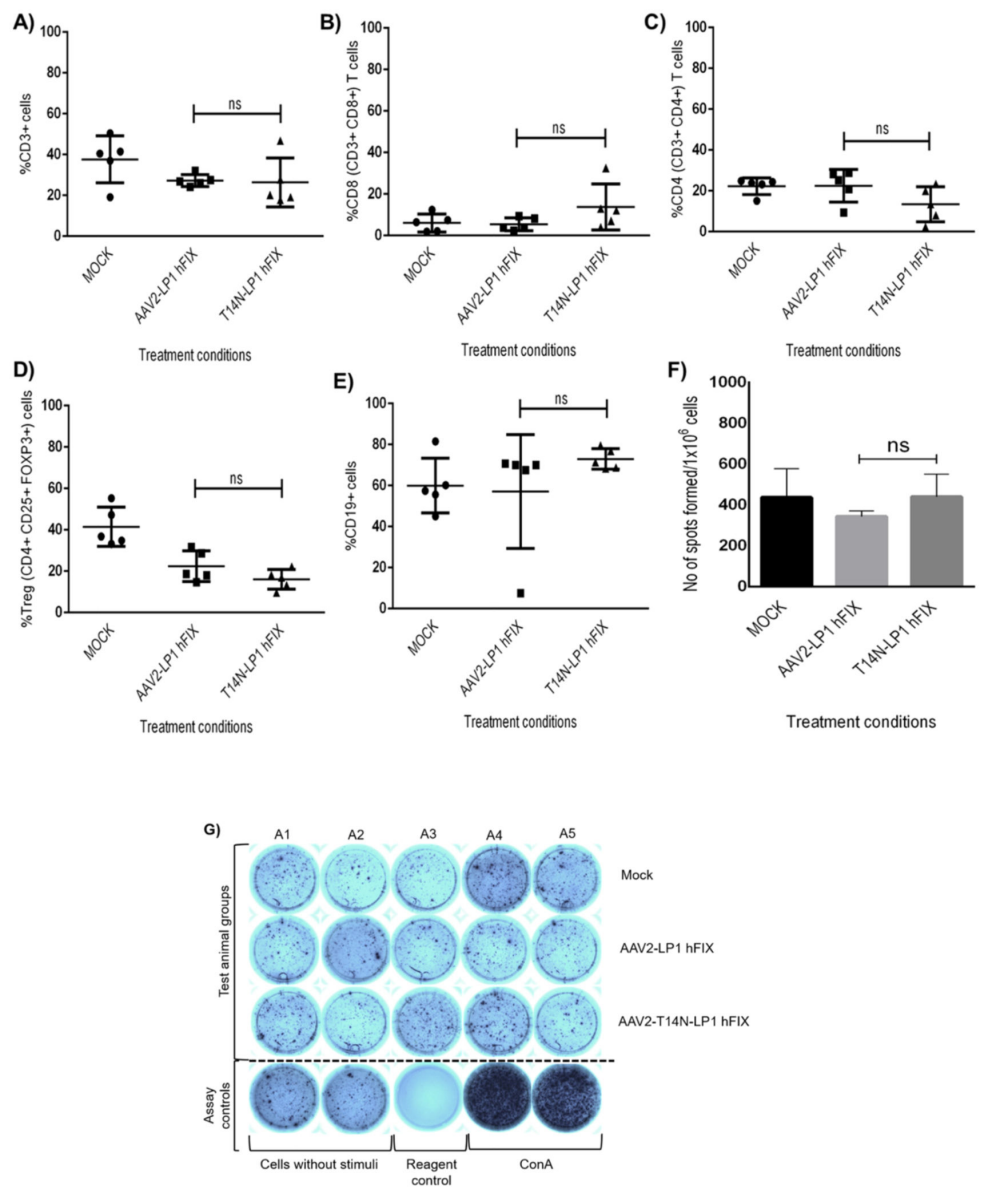
Evaluation of immune response in hemophilia B mice administered with AAV2-WT or AAV2-T14N mutant encoding human factor (F)IX transgene. Blood samples collected at 12 weeks after hepatic gene transfer was assessed for T-cell and B-cell markers by flow cytometry. Data for CD3+ T lymphocytes (A); cytotoxic T cells (CD3+CD8+) (B); and helper T cells (CD3+CD4+) (C); B-lymphocytes (E) from peripheral blood and regulatory T cells (CD4+CD25+ & FoxP3) in splenocytes (D) from mock-injected or AAV2-injected animals (*n* = 5) are shown. (F) Number of spots generated by 1 × 10^6^ splenocyte cells stimulated by the AAV2 capsid-specific peptide in the ELISPOT assay. (G) Representative images of the treatment groups, positive controls [concanavalin (Con) A], reagent control (without cells), cells without stimuli as blank controls, in ELISPOT plate are provided below. Data are shown as the mean ± standard deviation of the number of spots obtained from splenocytes seeded in duplicate wells for each of the five mice per group. *P* values were not significant (*p* > 0.05) vs WT-AAV2 injected hemophilia B mice.

To further understand if the mutant vectors alter the host immunogenic response, we measured the cytotoxic and helper T lymphocyte and B lymphocytes after FIX gene therapy. As can be seen in Figure 5, peripheral T lymphocyte (CD3+ CD4+ and CD8+) counts between AAV2-WT-hFIX and AAV2-T14N-hFIX administered groups were not significantly different. Similarly, the B-cell count among the mock (60 ± 13%) and the treated groups (57 ± 27% in AAV2 treated and 73 ± 5% in AAV2-T14N) was similar. Further quantitation of immune modulatory T regulatory cells (CD4+CD25+ and FoxP3+) in splenocytes indicated a similar finding. Taken together, our data demonstrate that AAV2-T14N vectors have a immunogenic response similar to WT-AAV2 vectors, which augurs well for its application in hepatic gene therapy.

To evaluate whether the AAV2 T14N vector alters capsid specific CD8+ T cell response, splenocytes were harvested from hemophilia B mice that received FIX gene therapy and evaluated by the ELISPOT assay. We did not observe any significant change in the IFN-*γ* activity between AAV2-LP1 hFIX-injected and T14N-LP1 hFIX-injected groups (*N* = 5/group) (Figure 5F,G). These data imply that the T14N mutation in the AAV2 capsid is not more immunogenic in comparison to AAV2-WT vectors in vivo.

### AAV2 Glycosylation Site-Modified Vectors Demonstrate a Higher EGFP Expression during Ocular Gene Transfer

**Figure 6:**
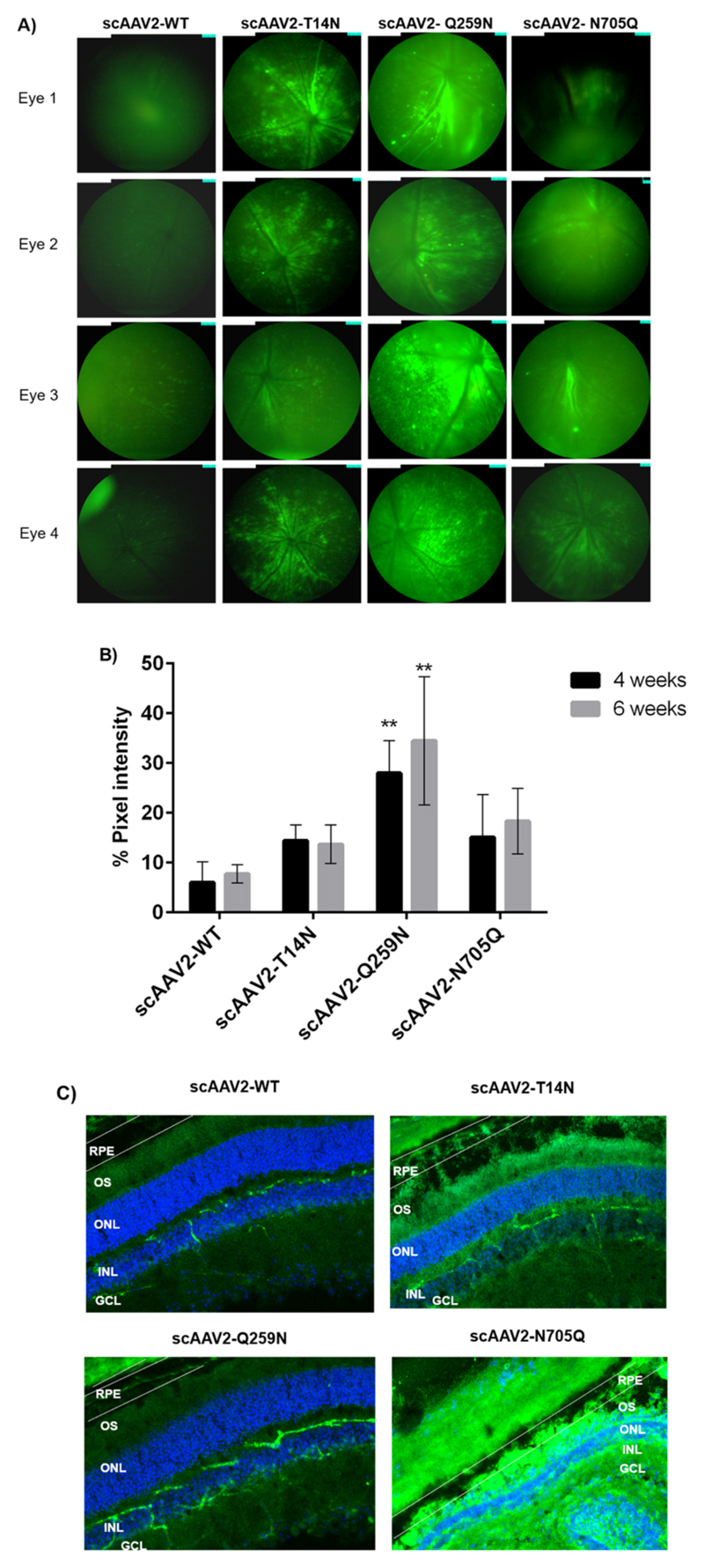
Ocular gene transfer efficiency of mutant AAV2 vectors. About 3 × 10^8^ vgs of either scAAV2-WT or AAV2 mutants were administered intravitreally into the eyes (*n* = 6) of C57BL6/J mice. Four and six weeks after ocular gene transfer, the gene expression was measured by fundus imaging in a Micron IV imaging system. Representative data of (A) 4 weeks after ocular gene transfer from scAAV2-EGFP, scAAV2-T14N, scAAV2-Q259N, and scAAV2-N705Q vector injected eyes are shown. The quantitative data of (A) are represented in (B), after image J analysis (*n* = 4). The fold difference in mean GFP intensity is provided in comparison to scAAV2-WT at 4 and 6 weeks. **p < 0.01 vs WT-AAV2-injected mice. (C) Immunohistochemical analysis of retina from C57BL6/J mice injected with AAV2 vectors. Retinal sections were probed by immunohistochemistry at 16 weeks after intravitreal administration of either PBS or scAAV2-WT or scAAV2-glyco-engineered vectors containing EGFP. GCL, ganglion cell layer; ONL, outer nuclear layer; INL, inner nuclear layer; OS, outer segment; RPE, and retinal-pigmented epithelium. Magnification is 200×.

To assess the transduction ability of the AAV2 mutants during ocular gene transfer, we performed intravitreal administration of scAAV2 vectors T14N, Q259N, and N705Q carrying EGFP into the eyes of C57BL6/J mice. Our data revealed an increased fluorescence in the retina of murine eyes injected with the glycosylation site modified vector, 4 and 6 weeks after administration (Figure 6A,B). This increase across 4–6 weeks was ~2.4 and 1.5× fold for AAV2-T14N; ~2.5 and 2.4-fold for AAV2-N705Q vector and ~4.6 and 4.4-fold for AAV2-Q259N vectors. To understand the pattern of GFP expression across the retina, we further performed immuno-histochemical studies. As can be seen in Figure 6C, a substantially higher EGFP expression was observed in the inner retinal layers, including the RPE layer, with the AAV2-T14N and N705Q vectors, whereas the AAV2-Q259N vector had a significantly higher expression in the inner nuclear layer (INL) of retina, in comparison to AAV2-WT vector-administered group. These data underscore the significant therapeutic potential of glycosylation site-modified AAV2 vectors for ocular gene therapy.

Further, characterization of AAV2-T14N vectors by fusion mass-spectrometry demonstrated the presence HexNacylation at residue N14 with Δmass of +203.08 Da. We subsequently performed functional characterization of AAV2-T14N vectors that were packaged with tunicamycin under glycosylation inhibited conditions or treated with glycosidase (PNGase F). Vectors were administered intravitreally at a dose of 2 × 10^8^ or 3 × 10^8^ vgs/eye, respectively. Two or four weeks later, these vectors demonstrated reduced transduction/permeation in the murine retina at different doses (Figure S7). This further suggested that the efficiency of the AAV2-T14N mutant vectors is due to the introduction of a capsid glycosylation site.

### Phenotypic Correction of Visual Function in *rd12* Mice

**Figure 7:**
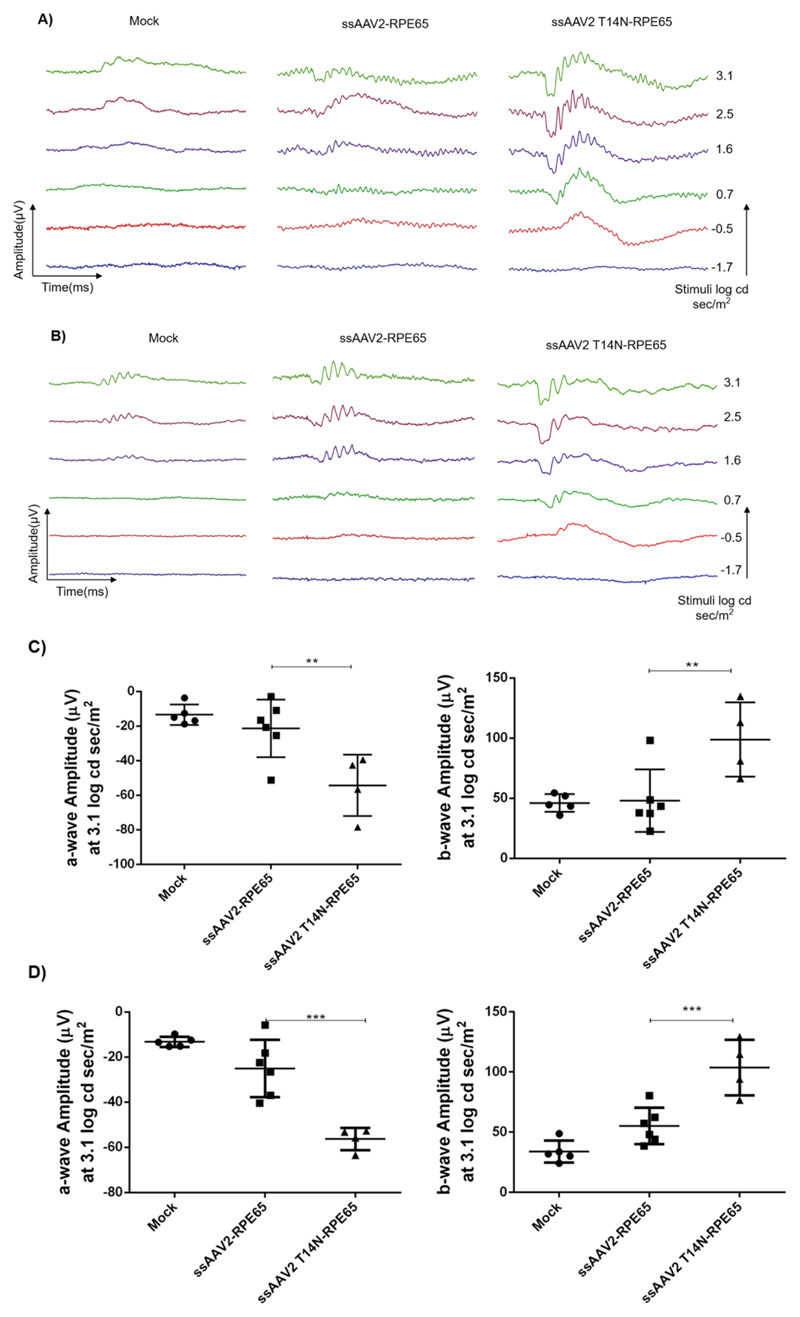
Visual function rescue in rd12 mice by AAV2 T14N mutant vector. (A) Representative images of the rescue in ERG wave forms in AAV2-T14N-injected eyes when compared to wild type vector injected and mock controls in rd12 mice after 6 weeks and (B) 10 weeks. A rescue in physiological vision as represented by the regain in qualitative wave form was noted. (C) Dot plot for “a wave” and “b wave” plotted against the mean amplitude obtained at 3.1 log cd s/m^2^ shows significant rescue in “a wave” form (left graph) and “b wave” (right graph) in the mutant vector injected group when compared to wild type vector injected and mock controls at 6 weeks and (D) 10 weeks post gene transfer. *n* = 4−6 eyes. Values represented are mean ± standard deviation. **p < 0.01, ***p < 0.001.

We packaged hRPE65 gene in AAV2-WT and AAV2-T14N capsids, since our data demonstrated that AAV2-T14N had consistently higher gene expression in liver (Figure 4) or superior permeability in retina up to the RPE layer (Figure 6). Subretinal administration of these vectors demonstrated that AAV2-T14N vectors rescued RPE65 gene function in a rd12 transgenic mouse with an impaired rod function. This is validated by the ERG analysis data shown in Figure 7. The analysis of the wave form and the resultant amplitude 6 and 10 weeks after gene transfer shows a significant increase in the “a wave” and “b wave” amplitude, denoting a significant correction in the photoreceptor response. Eyes that were administered with AAV2-T14N-hRPE65 vectors had an improved ERG profile when compared to mock (*p* = 0.002) and AAV2-WT-hRPE65-injected animals (*p* = 0.007) (*n* = 4−6 per group). Subsequently, similar studies with AAV2-Q259N-hRPE65 vectors (*n* = 4 per group) demonstrated an improvement in photoreceptor response 32 weeks after subretinal gene transfer at a low vector dose of 1 × 10^8^ vgs per eye (Figure S8).

## Discussion

Viruses are dependent on various host cellular factors to establish an infective life cycle. The typical events following a viral infection include the binding of the virus to the cellular receptors, its internalization, cytoplasmic trafficking, and nuclear entry, which require its interaction with a number of cellular factors. For example, AAV2 is known to interact with membrane-associated heparan sulfate proteoglycan and utilize it as the primary cellular receptor. Subsequently, several studies have reported other cellular factors including phosphorylation^9^ and ubiquitination^30^ that modulate AAV transduction, but very little data exist on other PTMs.

Given the versatility and diversity of the glycosylation as a PTM, it is not surprising that many studies have suggested that cellular glycosylation has a profound impact on the viral life cycle. Enveloped viruses (influenza and HIV virus) utilize the cellular glycosylation machinery for their entry, replication, and survival in the host.^31^ In the case of nonenveloped viruses, it has been reported that adenovirus contain glycosylated proteins, including a mono O-linked GlcNAc addition to the fiber protein.^32^ Similarly, glycosylation is known to be important for the correct folding of rotavirus capsid protein (VP7).^33^ The role of cellular glycosylation in the transduction process is known in the case of murine leukemia retrovirus, where inhibition of cellular glycosylation, enhanced its transduction by 200-fold, particularly in cells which were initially resistant to infection.^34^

In the case of AAV vectors, very minimal data exist on the role of cellular glycosylation and its impact on the virus. The identity and scope of glycan modifications to AAV capsid or the cellular proteins that impact AAV life cycle is important, as such modifications can be beneficial or detrimental during gene transfer. It is worthy to note that glycosylation modifications in other viruses have striking phenotypes—from masking the recognition by the antibody or leading to antibody recognition^31^ or impacting the viral intracellular trafficking process.^35^ In the present study, we performed global assays for studying the effect of glycosylation on AAV2 infection and in silico estimation of other capsid residues that may be potentially glycosylated.

In our first set of experiments, we estimated the regulation of AAV transduction in conditions of altered cellular glycosylation. To simplify the interpretation of outcome data, a scAAV2 serotype expressing a GFP reporter was used. We found that transduction efficiency of scAAV2 was increased by 30−74% in the presence of different glycosylation inhibitors in a highly permissive, HeLa cell line. Nonetheless, the quantum of increase in AAV2 transduction was variable according to the type of glycosylation inhibitor used. For example, pretreatment of tunicamycin enhanced AAV2 transduction by 74% whereas swainsonine-enhanced transduction by only 5%. These differences are likely due to the fact that these inhibitors target different cellular proteins/pathway involved in glycosylation. For example, tunicamycin acts on GlcNAc transferase while swainsonine is known to target *α*-mannosidase II proteins.^36^ The data generated here suggest that cellular glycosylation status is an important determinant of AAV2 transduction. The precise mechanism of increased transduction of scAAV2 because of the decreased glycosylation status needs to be investigated in detail, including alterations to cell surface receptors (heparan sulfate proteoglycan, HSPG) and by tracking the fate of the virus at multiple steps of its infection process, in relation to the major cellular glycosylation proteins and their networks.

Further, to assess if cellular glycosylation status impacts virus production, we packaged AAV2 in the presence of glycosylation modulators. Our results demonstrate that the yield of viral particles was reduced by up to 1-fold in comparison to packaging cells that were not treated with any pharmacological agent. It has been reported that in the presence of cellular glycosylation inhibitors such as tunicamycin, the yield of adenovirus is reduced due to the inhibition of viral DNA replication.^37^ Similarly, an activator of glycosylation such as ATRA is known to modulate replication of HIV or herpes simplex virus.^38,39^ It is possible that the reduced AAV vector yield in the presence of glycosylation modifiers noted here, could be because of altered functionality of cellular proteins necessary for virus packaging or by alterations to viral *Rep* and *helper* proteins that participate in AAV DNA replication. Interestingly, Nash et al. have identified 188 cellular proteins from 16 different functional categories which interact with AAV rep protein, known to be involved in replication, control of viral and cellular transcription and protein translation.^40^ Several of these proteins associated with AAV2 *Rep* (such as RCN1 and IRS4) are known to be heavily glycosylated.^41^

To understand if modification of predicted glycosylation sites on AAV2 capsid could enhance the outcome of gene transfer, a total of 24 glycosylation site-modified vectors were developed of which a few demonstrated increased transduction in vitro and in vivo. Four (A204T, K314T, Y377N, and N335Q) of 24 AAV2 mutants resulted in a reduced vector yield, which may be due to the mutation in the overlapping region between assembly-activating protein (AAP) and VP1. The mutations in the AAP region are likely to impact the capsid assembly process. Of the five capsid mutants developed by targeting O-linked glycosylation motifs, only a fraction (*n* = 2/5), namely, the S149A and S207A mutants in which potential O-linked glycosylation sites were abolished, exhibited increased transduction in the order of ~44−50% in ARPE-19 cells, while generation of O^+^ mutants did not improve the transduction of AAV2-EGFP vectors. Similarly, of the nineteen N-glycosylation site mutant capsids developed, four (N^+^ mutants: T14N, Q259N and S412N and N^−^ mutant: N705Q) had enhanced transduction in multiple cell lines. Taken together, these studies highlight that in general, modification of N-linked sites had relatively higher transgene expression from AAV2 vectors. Interestingly, the nature and type of glycosylation site modification did not yield a distinctive phenotype of gene expression. For example, both N^+^ [T14N] and N^−^ mutants [N705Q] had increased transduction. On the other hand, N^+^ mutants (E499N, G504N, R513T, Q677N) and a N^−^ mutant, N382Q were noninfectious. This shows a considerable heterogeneity in the phenotypic expression of the mutants. A similar phenomenon has been noted with tyrosine or serine/threonine or lysine mutants.^9,19^ Thus, it stands to reason that the efficacy of any AAV capsid mutant generated is sequence-, context-, and cell-type-specific. Enhanced transduction efficiency of the mutant vectors could be also possibly due to strong interaction with cell surface receptors, which remains to be elucidated (AAVR or HSPG).^42,43^ Interestingly, among all the mutants generated, only one N-glycosylation site abolished mutant (N705Q) demonstrated a partial immune escape to Nabs. This residue is within a known B-cell epitope recognition sequence and is experimentally verified in a murine model.^44^ However, it is possible that a single site modification of the immune epitope may not be sufficient to disrupt the interaction of the epitope with the pooled immunoglobulin (IVIG), which is of polyclonal origin.

We further reasoned that adaptation of the glycosylation site-modified capsid to test the expression of the therapeutic genes with or without cell-specific promoters for the desired tissue tropism, will provide further insights into its therapeutic potential. We utilized the most consistent mutant vector in our in vitro studies, AAV2-T14N to package hFIX under the control of a liver specific promoter, LP1. T14N capsid, improved the circulating levels of hFIX consistently by 2-fold in hemophilia B mice at 4, 10, and 12 weeks after hepatic gene transfer. Our experiments also demonstrated no significant difference in T-cell and B-cell populations between WT-AAV2-treated and T14N-treated hemophilia B mice. These findings suggest that it should be possible to obtain a similar level of therapeutic hFIX expression with a significantly lower dose of the AAV2 T14N vectors. To further test the efficacy of glycosylation site-modified vector within a second organ, we delivered EGFP containing AAV2-T14N, Q259N, and N705Q vectors, intravitreally into the eyes of C57BL6/J mice. At a significantly reduced dose (3 × 10^8^ vgs), we observed a 2 to 4-fold increase in GFP expression for mutants, N705Q, Q259N and enhanced permeation of T14N vectors, respectively. Further mass spectrometric analysis of the AAV2-T14N vector revealed a HexNacylation modification. The functional characterization of AAV2-T14N vectors showed an altered retinal permeation and a reduction in gene expression from these vectors when treated with PNGase F or when packaged in the presence of glycosylation inhibitors. These data confirmed the presence of a glycan modification in the T14N site (Figure S7). Our findings suggest that such glycosylation site-modified mutant capsids may be excellent candidates to test the efficacy of the therapeutic gene, hRPE65 in a transgenic model of LCA2. Thus, subretinal administration of these vectors, such as AAV2-T14N and AAV2-Q259N containing RPE65 gene, substantially rescued the visual parameters in rd12 mice. The dose at which this phenotypic rescue was observed is significantly lower (1 × 10^8^ to 7 × 10^8^ vgs/eye) than reported in other studies.^45–47^

## Conclusions

Our study has generated glycosylation site-modified AAV2 vectors that demonstrate significant therapeutic potential for ocular and hepatic gene therapy applications.

## Supplementary Material

The Supporting Information is available free of charge on the ACS Publications website at DOI: 10.1021/acs.molpharmaceut.9b00959.

List of glycosylation modulators used in cell lines during vector transduction and packaging; list of primers used for AAV2 mutant generation; primer sequences used for human coagulation factor IX transcript analysis; list of AAV2 mutants predicted by in silico prediction analysis; list of AAV2 vectors generated and their physical particle titers; gating strategy for enumeration of helper and cytotoxic T cells; effect of cellular glycosylation modulators on AAV2 packaging; schematic representation of AAV2 VP1 capsid with sites of N-linked glycosylation motifs and O-linked glycosylation motifs; targets for glycosylation site modification within the putative immune epitopes of AAV2 capsid; neutralization antibody assay; human coagulation factor IX expression from AAV2 vectors in a hepatic cell line; characterization AAV2-T14N vectors: mass spectrometric analysis of glycosylation in the capsid and their retinal permeation characteristic when packaged in the presence of tunicamycin or when treated with a glycosidase; and visual function in rd12 mice after administration of AAV2 Q259N vectors (PDF)

Tables S1-S5, Figures S1-S8

## Abbreviations

AAV: adeno-associated virus
PTM: post-translational modification
MOI: multiplicity of infection
hFIX: human coagulation factor IX
LCA: Leber congenital amaurosis
IVIG: intravenous immunoglobulins
