# Supplementary material for: Molecular Engineering of Adeno-Associated Virus Capsid Improves Its Therapeutic Gene Transfer in Murine Models of Hemophilia and Retinal Degeneration
Source: Mol Pharm. Author manuscript; Available in PMC 2020 Feb 21. (PMC7035104; doi:10.1021/acs.molpharmaceut.9b00959)
Supplement: Tables S1-S5, Figures S1-S8 [file EMS85813-supplement-Tables_S1_S5__Figures_S1_S8.pdf]

Supporting Information

Tables

Table S1: List of glycosylation modulators used in cell lines during vector transduction and packaging.

Glycosylation Modulator	Role	Concentration Used	Incubation time period
Tunicamycin	N-linked inhibitor[1-2]	0.125 µg/ml	24 hours
Swainsonine	N-linked inhibitor[1]	6 µg/ml	24 hours
Benzyl-α-GalNAc	O- linked inhibitor[3]	1.5 mM	24 hours
Alloxane	O-linked inhibitor[4]	5 mM	12 hours
All-trans-retinoic-acid	Glycosyltransferase Activity[5]	1 µM	48 hours

Table S2: List of primers used for AAV2 mutant vector generation. The nucleotides which are mutated in reference to wildtype AAV2 sequence [NC_001401.2] are in **bold** font.

AAV2 mutant	Primers used
AAV2 S149A	5'-AAAGAGGCCGGTAGAGCAC**GCT**CCTGTGGAGCCAGACT-3'
	5'-AGTCTGGCTCCACAGG**AGC**GTGCTCTACCGGCCTCTTT-3'
AAV2 S207A	5'-TAATACGATGGCTACAGGC**GCT**GGCGCACCAATGGCAGACAAT-3'
	5'-ATTGTCTGCCATTGGTGCGCC**AGC**GCCTGTAGCCATCGTATTA-3'
AAV2 A193S	5'-GGACAGCCACCA**TCA**GCCCCCTCTG-3'
	5'-CAGAGGGGGC**TGA**TGGTGGCTGTCC-3'
AAV2 A204T	5'-GGGAACTAATACGATG**ACT**ACAGGCAGTGGCG-3'
	5'-CGCCACTGCCTGT**AGT**CATCGTATTAGTTCCC-3'
AAV2 A655T	5'-CAAGAACACCCCGGTACCT**ACG**AATCCGTCGACCA-3'
	5'-TGGTCGACGGATT**CGT**AGGTACCGGGGTGTTCTTG-3'
AAV2 T14N	5'-TCTTCCAGATTGGCTCGAGGAC**AAT**CTCTCTGAAGGAATAAG-3'
	5'-CTTATTCCTTCAGAGAG**ATT**GTCCTCGAGCCAATCTGGAAGA-3'
AAV2 E216T	5'-ACCAATGGCAGACAATAAC**ACG**GGCGCCGACGGAGTGGGTAA-3'
	5'-TTACCCACTCCGTCGGCGCC**CGT**GTTATTGTCTGCCATTGGT-3'
AAV2 Q259N	5'-ACAACAACCACCTCTACAAA**AAC**ATTTCCAGCCAATCAGGAGC-3’
	5'-GCTCCTGATTGGCTGGAAAT**GTT**TTTGTAGAGGTGGTTGTTGT-3'
AAV2 K314T	5'-CCGACCCAAGAGACTCAACTTC**ACG**CTCTTTAACATT-3'
	5'-AATGTTAAAGAG**CGT**GAAGTTGAGTCTCTTGGGTCGG-3'
AAV2 Q319T	5'-GACTCAACTTCAAGCTCTTTAACATT**ACG**GTCAAAGAGGTCACGCAGAATGAC-3'
	5'-GTCATTCTGCGTGACCTCTTTGAC**CGT**AATGTTAAAGAGCTTGAAGTTGAGTC-3'
AAV2 Y377N	5'-CATGGTGCCACAGTATGGA**AAC**CTCACCCTGAACAACGG-3'
	5'-CCGTTGTTCAGGGTGAG**GTT**TCCATACTGTGGCACCATG-3'
AAV2 G383T	5'-CCTCACCCTGAACAAC**ACG**AGTCAGGCAGTAGGA-3'

	5'-TCCTACTGCCTGACT**CGT**GTTGTTCAGGGTGAGG-3'
AAV2 S412N	5'-CGGAAACAACTTTACCTTC**AAC**TACACTTTTGAGGACGTTC-3'
	5'-GAACGTCCTCAAAAGTGTA**GTT**GAAGGTAAAGTTGTTTCCG-3'
AAV2 S446N	5'-CATCGACCAGTACCTGTATTACTTA**AAC**AGAACAAACACTCCAAGTGG-3'
	5'-CCACTTGGAGTGTTTGTTCT**GTT**TAAGTAATACAGGTACTGGTCGATG-3'
AAV2 E499N	5'-CTGCAGATAACAACAACAGT**AAT**TACTCGTGGACTGGAGCTAC-3'
	5'-GTAGCTCCAGTCCACGAGTA**ATT**ACTGTTGTTGTTATCTGCAG-3'
AAV2 G504N	5'- ACAACAGTGAATACTCGTGGACT**AAC**GCTACCAAGTACCACCTCAA -3'
	5'- TTGAGGTGGTACTTGGTAGC**GTT**AGTCCACGAGTATTCACTGTTGT -3'
AAV2 R513T	5'-GTACCACCTCAATGGC**ACG**GACTCTCTGGTGAATCC-3'
	5'-GGATTCACCAGAGAGTC**CGT**GCCATTGAGGTGGTAC-3'
AAV2 Q677N	5'-CTTCATCACACAGTATTCCACGGGA**AAC**GTCAGCGTGGAGATC-3'
	5'-GATCTCCACGCTGAC**GTT**TCCCGTGGAATACTGTGTGATGAAG-3'
AAV2 N223Q	5'-GCCGACGGAGTGGGT**CAA**TCCTCAGGAAATTGGCATTGC-3'
	5'-GCAATGCCAATTTCCTGAGGA**TTG**ACCCACTCCGTCGGC-3'
AAV2 N335Q	5'-CGACGACGATTGCCAAT**CAG**CTTACCAGCACGGTTCA-3'
	5'-TGAACCGTGCTGGTAAG**CTG**ATTGGCAATCGTCGTCG-3'
AAV2 N382Q	5'-ATACCTCACCCTGAAC**CAG**GGGAGTCAGGCAGTAG-3'
	5'-CTACTGCCTGACTCCC**CTG**GTTCAGGGTGAGGTAT-3'
AAV2 N495/6/7Q	5'-GCGAGTATCAAAGACATCTGCAGAT**CAGCAGCAG**AGTGAATACTCGTGGACTGGAGC-3'
	5'-GCTCCAGTCCACGAGTATTCACT**CTGCTGCTG**ATCTGCAGATGTCTTTGATACTCGC-3'
AAV2 N703/5Q	5'-GGAATCCCGAAATTCAGTACACTTCC**CAG**TAC**CAG**AAGTCTGTTAATGTAGACTTTACTGT-3'
	5'-ACAGTAAAGTCTACATTAACAGACTT**CTG**GTA**CTG**GGAAGTGTACTGAATTTCGGGATTCC-3'
AAV2 N705Q	5'-CCCGAAATTCAGTACACTTCCAACTAC**CAG**AAGTCTGTTAATGTAGACTTTACTG-3'
	5'-CAGTAAAGTCTACATTAACAGACTT**CTG**GTAGTTGGAAGTGTACTGAATTTCGGG-3'

Table S3: Primers used for human coagulation factor IX transcript analysis.

S.No	PRIMER NAME	SEQUENCE
1	B-Actin	5'-AGTCCCTTGCCATCCTAAAAG-3'
		5'-CAATGCTATCACCTCCCCTG-3'
2	Human FIX	5'-CATGGAGGAGAAGTGCAGCTTG-3'
		5'-ATGTTGCAGGTCACATCCAGCTC-3'

Table S4: List of AAV2 mutants predicted by *in-silico* prediction analysis. Glycosylation site modified vectors were generated by introducing mutations at specific residues to create or abolish glycosylation motifs. Glycosylation potential of the modified sites are represented after an *in silico* analysis.

S.No	Mutant Type	Mutant Name	Mutation residue	Glycosylation motif change	Glycosylation potential
1	**O⁻**	AAV2 S149A	S→A (TCT→GCT)	HSPV→HAPV (148 - 151)	0.9346→0
2		AAV2 S207A	S→A (AGT→GCT)	GSGA→GAGA (206 - 209)	08098→0
3	**O⁺**	AAV2 A193S	A→S (GCA→TCA)	PAAP→PSAP (192 - 195)	0→0.7562
4		AAV2 A204T	A→T (GCT→ACT)	MATG→MTTG (203 - 206)	0→08928
5		AAV2 A655T	A→T (GCG→ACG)	PANP→PTNP (654 - 657)	0→0.5295
6	**N⁺**	AAV2 T14N	T→N (ACT→AAT)	TLSE→NLSE (14 - 17)	0→0.7353
7		AAV2 E216T	E→T (GAG→ACG)	NNEG→NNTG (214 - 217)	0.6836→0.6912
8		AAV2 Q259N	Q→N (CAA→AAC)	QISS→NISS (259 - 262)	0→0.7022
9		AAV2 K314T	K→T (AAG→ACG)	NFKL→NFTL (312 - 315)	0.8122→0.8264
10		AAV2 Q319T	Q→T (CAA→ACG)	NIQV→NITV (317 - 320)	0.68→0.70
11		AAV2 Y377N	Y→N (TAC→AAC)	YLTL→NLTL (377- 380)	0→0.7701
12		AAV2 G383T	G→T (GGG→ACG)	NNGS→NNTS (381 - 384)	0→0.7929
13		AAV2 S412N	S→N (AGC→AAC)	SYTF→NYTF (412 - 415)	0→0.65
14		AAV2 S446N	S→N (AGC→AAC)	SRTN→NRTN (446 - 449)	0→0.7364
15		AAV2 E499N	E→N (GAA→AAT)	EYSW→NYSW (499 - 502)	0→0.6239
16		AAV2 G504N	G→N (GGA→AAC)	GATK→NATK (504 - 507)	0→0.7456
17		AAV2 R513T	R→T (AGA→ACG)	NGRD→NGTD (511 - 514)	0.633→0.655
18		AAV2 Q677N	Q→N (CAG→AAC)	QVSV→NVSV (677 - 680)	0→0.7119
19	**N⁻**	AAV2 N223Q	N→Q (AAT→CAA)	NSSG→QSSG (223 - 226)	0.5291→0
20		AAV2 N335Q	N→Q (AAC→CAG)	NLTS→QLTS (335 - 338)	0.7998→0
21		AAV2 N382Q	N→Q (AAC→CAG)	NGSQ→QGSQ (382 - 386)	0.6139→0
22		AAV2 N495/6/7Q	N→Q (AAC→CAG)	NNNS→QQQS (495 - 498)	0.3056→0

23		AAV2 N703/5Q	N→Q (AAC→CAG)	NYNK→QYQK (703 - 705)	0.7014→0
24		AAV2 N705Q	N→Q (AAC→CAG)	NKSV→QKSV (705 - 708)	0.7014→0

Table S5: List of AAV2 vectors generated and their physical particle titers.

Vector	Titre (vgs/ml)
AAV2 EGFP	4.18e+11
AAV2 S149A	5.34e+11
AAV2 S207A	2.72e+11
AAV2 193S	8.79e+11
AAV2 A204T	2.37e+10
AAV2 A655T	2.03e+11
AAV2 T14N	2.80e+11
AAV2 E216T	1.08e+11
AAV2 Q259N	3.18e+11
AAV2 K314T	1.03e+10
AAV2 Q319T	5.04e+11
AAV2 Y377N	2.81e+09
AAV2 G383T	1..47e+11
AAV2 S412N	3.91e+11
AAV2 S446N	8.31e+12
AAV2 E499N	3.75e+11
AAV2 G504N	1.33e+11
AAV2 R513T	4.51e+11
AAV2 Q677N	2.64e+11
AAV2 N223Q	1.36e+11
AAV2 N335Q	8.35e+09
AAV2 N382Q	1.59e+11
AAV2 N495/6/7Q	2.20e+11
AAV2 N703/5Q	3.87e+11
AAV2 N705Q	4.80e+11

Supporting information

Figures

Figure S1: Gating strategy for enumeration of Helper and Cytotoxic T cells. Lymphocyte population was gated from PBMCs and CD3+ cells were counted, further CD3+ gate was applied to count CD3+ CD4+ (Helper T cells) and CD3+ CD8+ (Cytotoxic T Cells) by flow cytometry [BD Accuri plus, BD Biosciences].

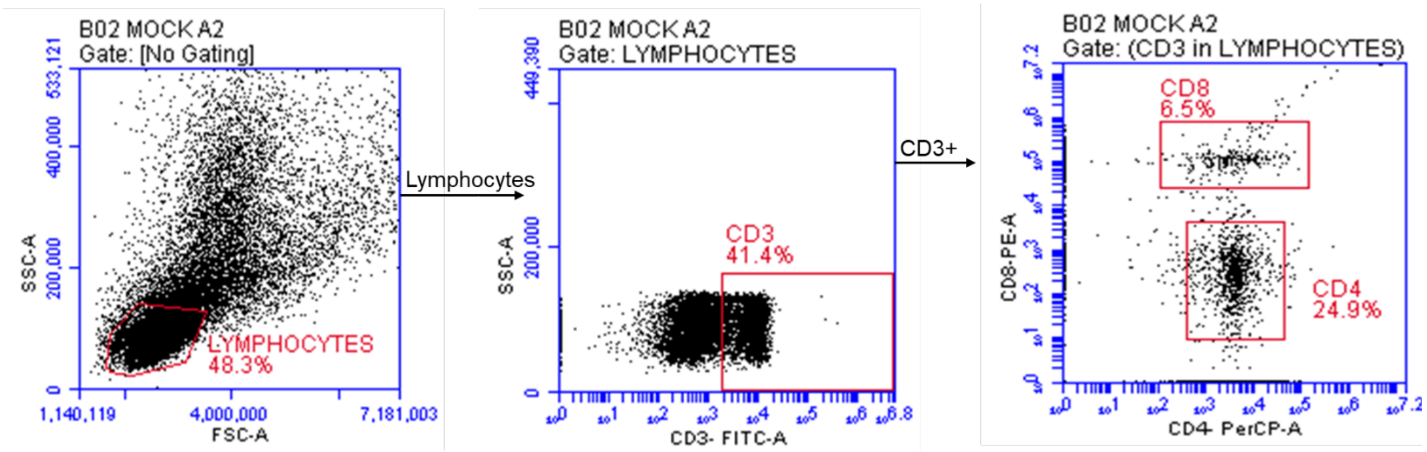

Figure S2: Effect of cellular glycosylation modulators on AAV2 packaging. A) scAAV2-EGFP vectors were packaged in AAV293 cells pretreated with (and during transfection) or without glycosylation modulators. Seventy-two hours post transfection of the packaging plasmids, viral particles were purified and titrated by a quantitative PCR. The data depicted are mean of two independent titration assays. The viral titers obtained after packaging are plotted as vector genomes/ml and are shown in log scale B) A similar assay performed in AAV293 cells which were treated with the drugs 6 hours post triple plasmid transfection. *$p<0.05$ vs. titers obtained in the absence of glycosylation modulators. Bars represent SD of the mean (n=6). *$p<0.05$ vs cells infected with scAAV2-EGFP vectors alone.

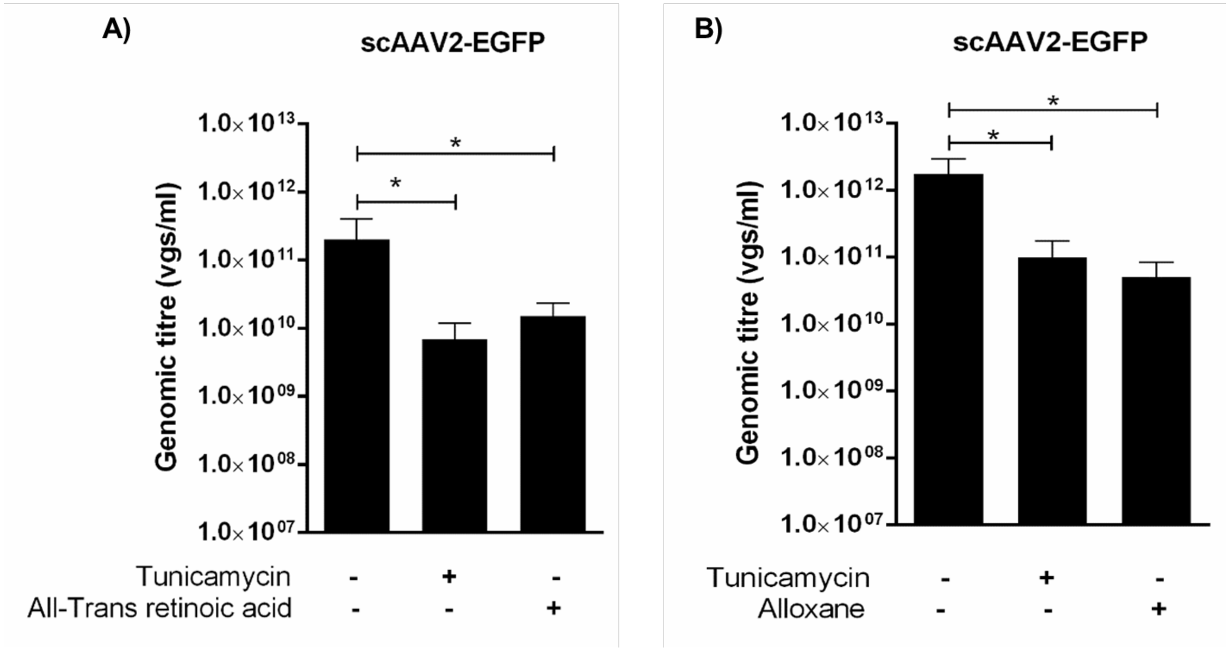

Figure S3: Schematic representation of AAV2 VP1 capsid with sites of N-linked glycosylation motifs (Blue font) and O-linked glycosylation motifs (Green font).

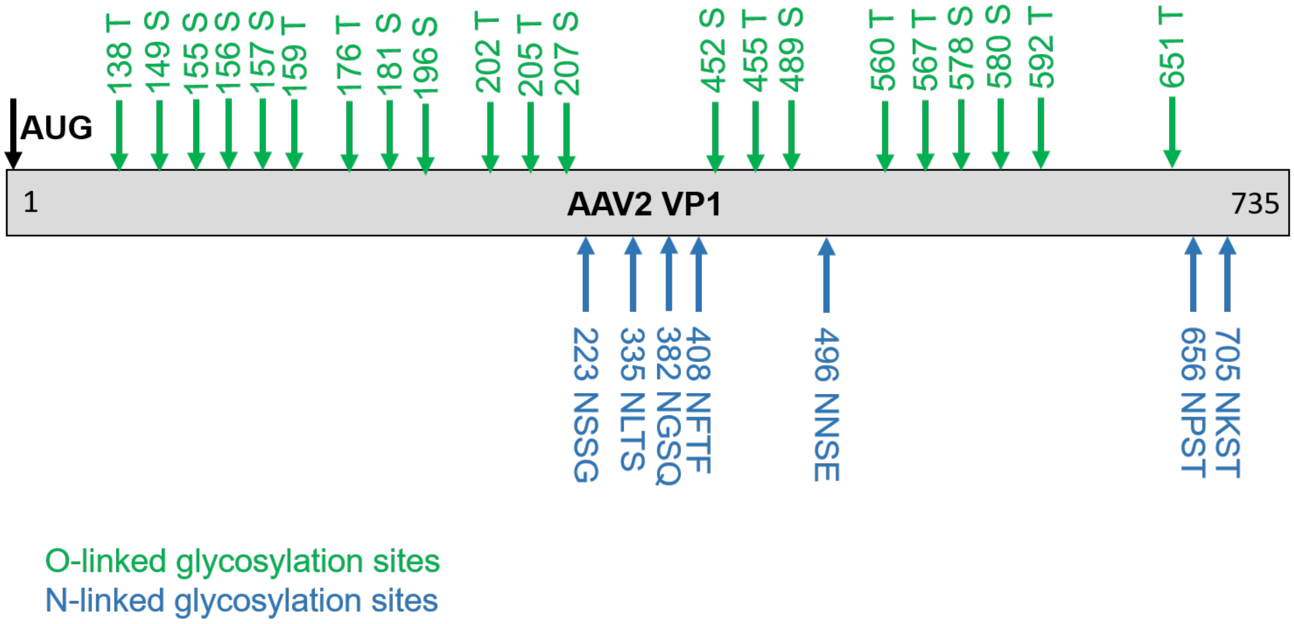

Figure S4: Targets for glycosylation site modification within the putative immune epitopes of AAV2 capsid. Sequence of the AAV2 capsid protein VP1 region with the predicted B-cell and T-cell immune epitope regions (red font), loop regions (highlighted in yellow) and endogenous N-glycosylation motif (blue font– *N-X-S/T residues*) and O-glycosylation motif (green font–*S/T residues*) is shown. In the absence of any prior data in AAV and since both addition and abolition of glycosylation sites are known to impart immune escape functions to other viruses, we strategized to either create new glycosylation sites within the predicted immunogenic epitopes or abolish endogenous glycosylation sites and study their effect further. Thus, residues chosen to introduce N glycosylation motif are represented with N+ symbol (n=13); sites chosen to abolish N glycosylation motifs are represented with N- symbol (n=6); sites chosen to introduce O glycosylation motif are represented with O+ symbol (n=3) and sites where potential O glycosylation sites were abolished are represented with O- symbol (n=2).

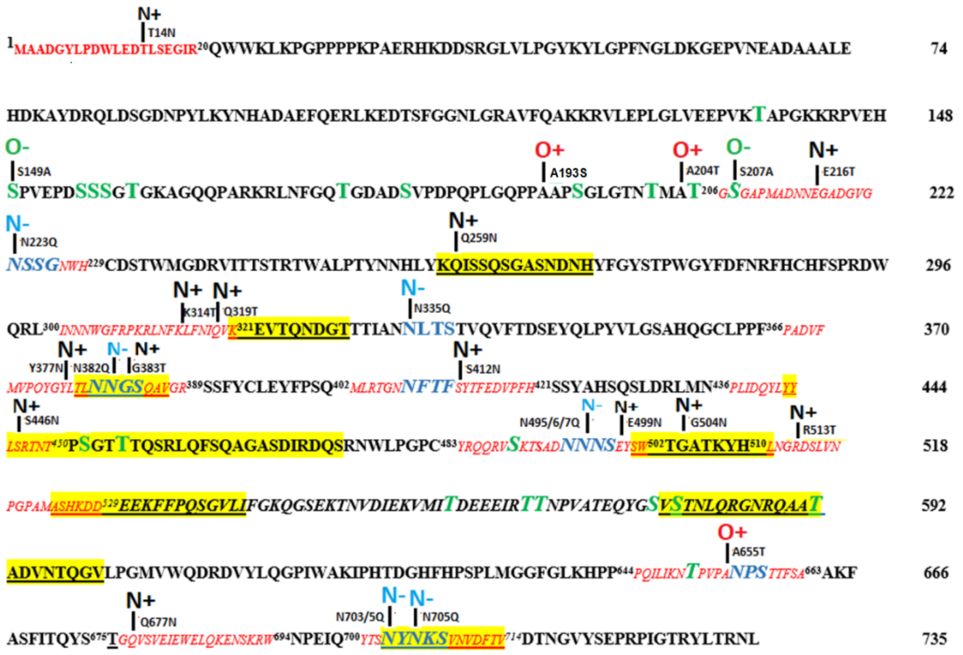

Figure S5: Neutralization antibody assay. The transduction potential of AAV2-WT vector expressing EGFP was assessed in the presence of different concentrations of intravenous immunoglobulin [IVIG]. The NAb titer is the highest IVIG dilution that inhibited AAV transduction of HeLa cells by 50% or more, compared to cells infected with AAV2 alone.

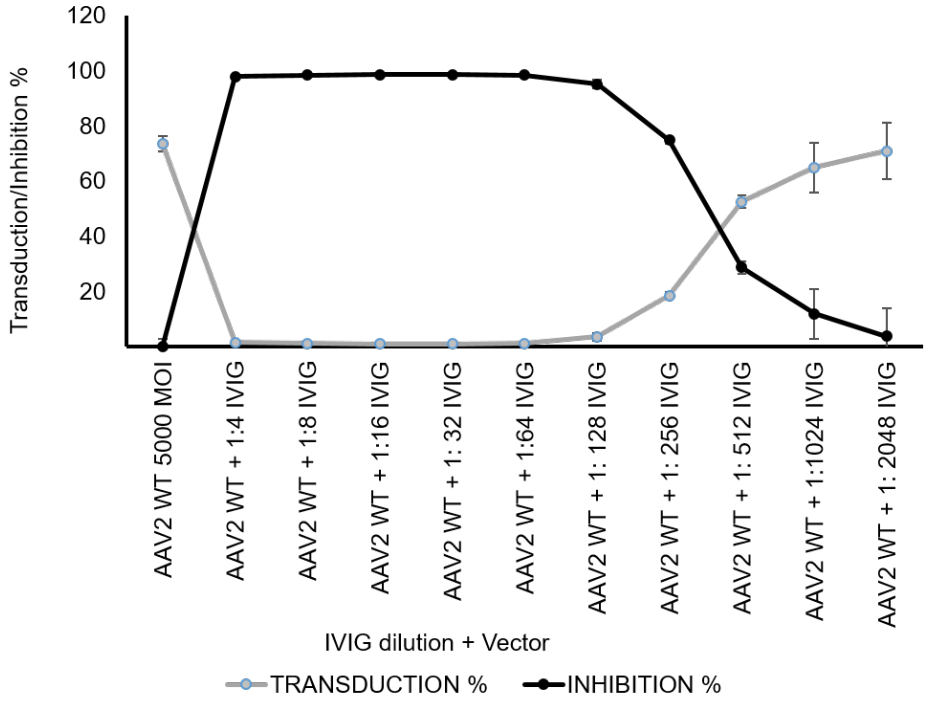

Figure S6: Human coagulation factor IX expression from AAV2 vectors in a hepatic cell line. The wildtype or mutant AAV2 vectors containing hFIX were infected at a MOI of $5x10^4$ vgs in Huh7 cells. Two days later, the hFIX transcript levels were assessed by quantitative PCR. Data shown is from 2 independent biological replicates with three technical replicates (n=6) for each condition. *p <0.05, **p <0.01 in comparison to FIX expression from cells infected with AAV2-WT vectors.

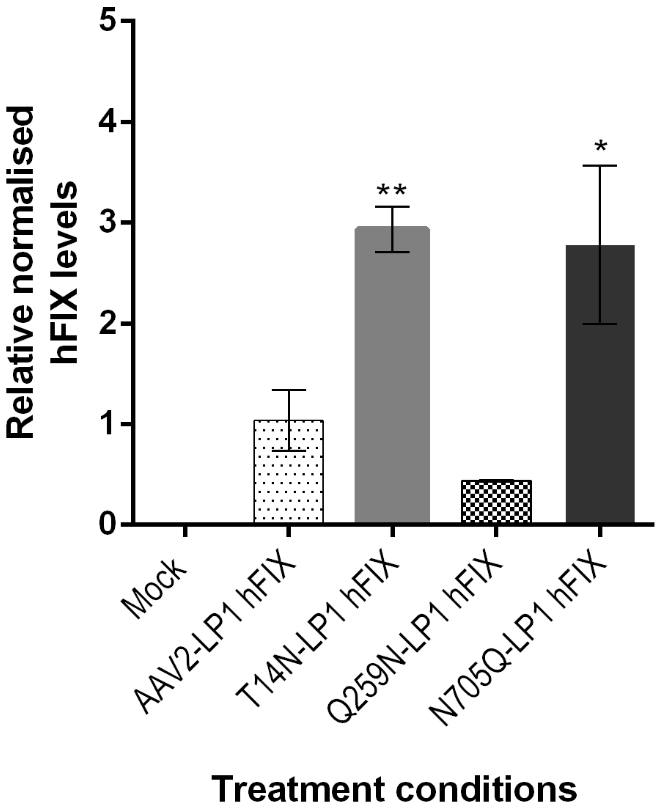

Figure S7: Characterization AAV2-T14N vectors: Mass spectrometric analysis of glycosylation in the capsid and their retinal permeation characteristic when packaged in the presence of Tunicamycin or when treated with a glycosidase. A) Representative spectra identified at N14 residue in AAV2-T14N vector. Mass spectrometry data of trypsin/Lys-C digested peptides from AAV2-T14N was acquired on an Orbitrap and analyzed by PEAKS X software. B) AAV2-T14N vectors packaged under cellular glycosylation inhibited condition, in the presence of Tunicamycin ($2x10^8$ vgs) or C) treated with PNGase F enzyme (250U) overnight at 37°C under non-denaturing condition ($3x10^8$ vgs) were intravitreally administered into C57BL6/J mice (n=4-5 eyes per group). Two or four weeks after vector delivery, the retinal sections were imaged by confocal microscopy to assess transgene (EGFP) expression and the permeation of the vector across the neural retina. Untreated AAV2-T14N vector was used as control for comparison. Representative images from three eyes for B) AAV2-T14N vectors packaged in the presence or absence of tunicamycin and C) AAV2-T14N vectors treated with or without PNGase F is shown. Mean fluorescence intensity was from animals administered with vectors packaged in presence of tunicamycin (N=4 eyes/group; 3 sections/eye) (D) or vectors treated with PNGase F (N=5 eyes/group; 3 sections/eye) (E) are shown. GCL, ganglion cell layer; ONL, outer nuclear layer; INL, inner nuclear layer; OS, outer segment; RPE, retinal pigmented epithelium. ***p<0.001 vs control AAV2T14N; Magnification-400X.

A)

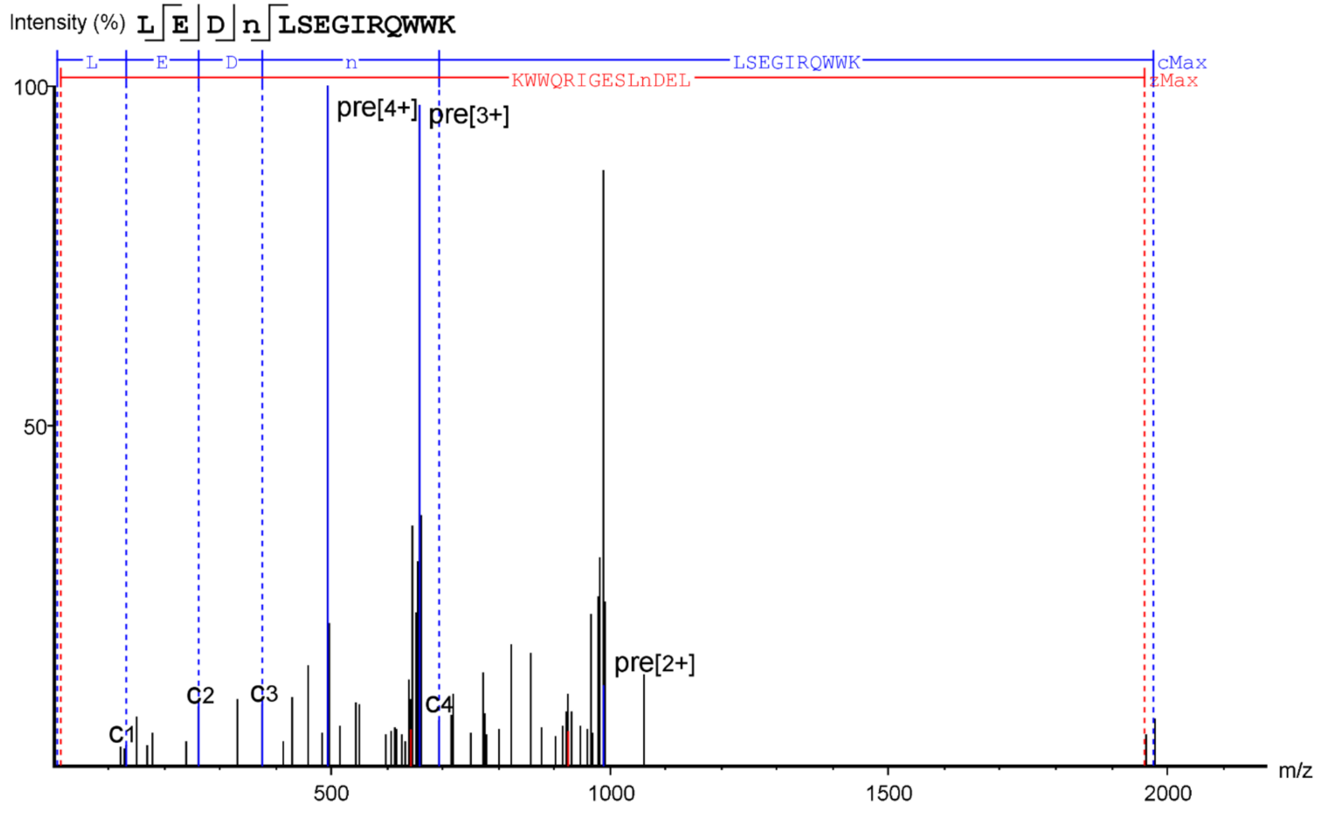

Glyco-engineered vector	Site	Modification	Peptide sequence (Δmass)
AAV2-T14N	N14	HexNacylation	LEDN(+203.08)LSEGIRQWWK

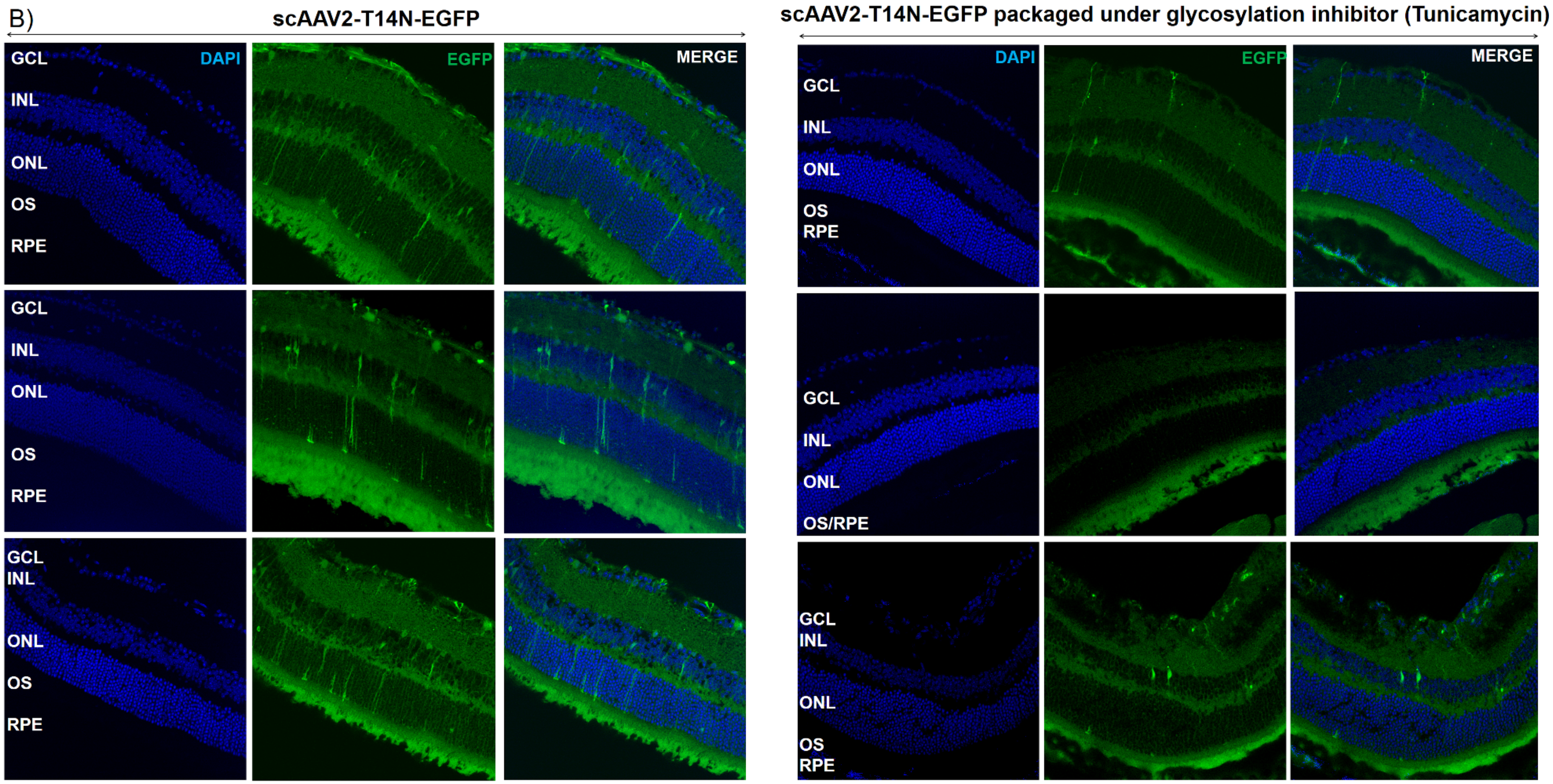
B)
scAAV2-T14N-EGFP
scAAV2-T14N-EGFP packaged under glycosylation inhibitor (Tunicamycin)
DAPI
EGFP
MERGE
GCL
INL
ONL
OS
RPE
OS/RPE

C)

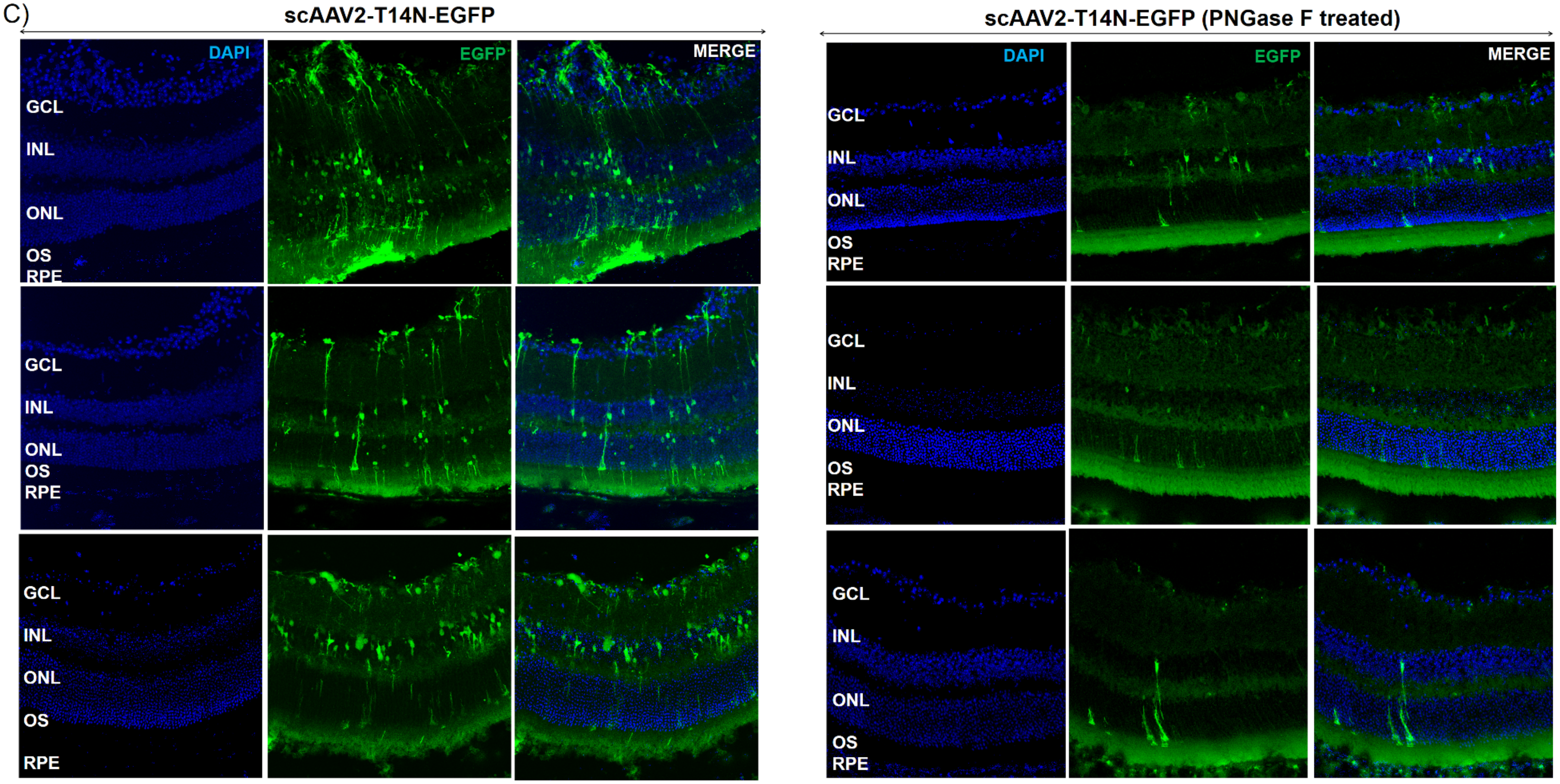
scAAV2-T14N-EGFP
scAAV2-T14N-EGFP (PNGase F treated)
DAPI
EGFP
MERGE
GCL
INL
ONL
OS
RPE

D) E)

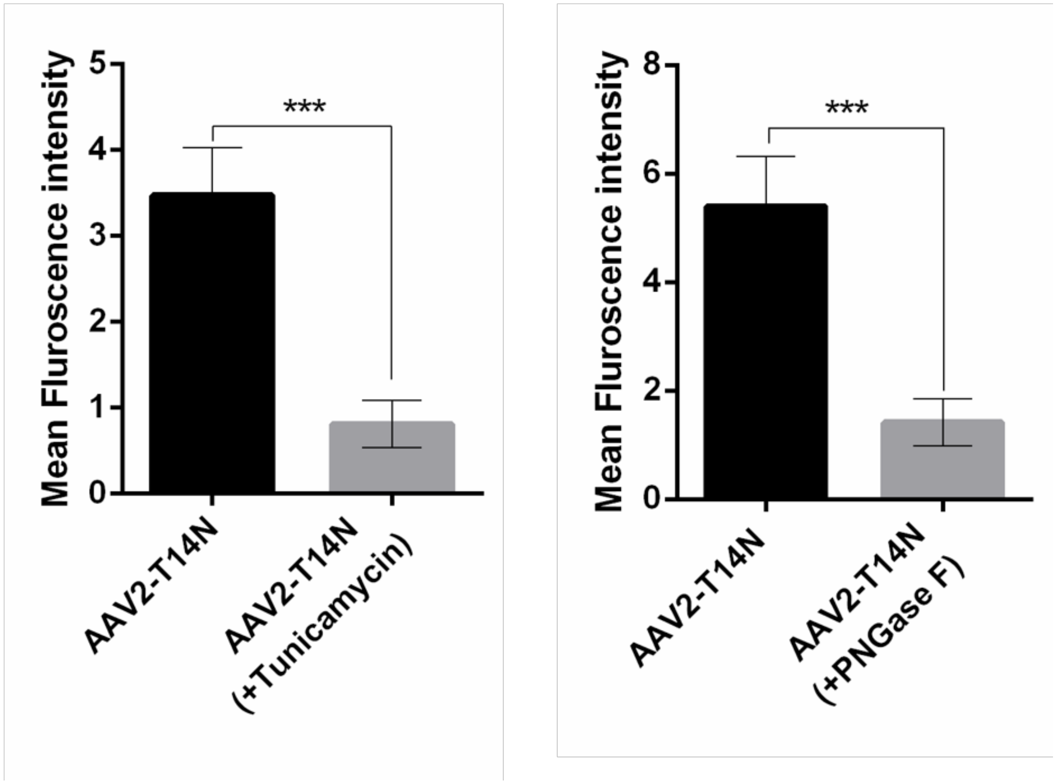

Mean Fluroscence intensity
5
4
3
2
1
0
***
AAV2-T14N
AAV2-T14N (+Tunicamycin)
Mean Fluroscence intensity
8
6
4
2
0
***
AAV2-T14N
AAV2-T14N (+PNGase F)

Figure S8: Visual function in rd12 mice after administration of AAV2 Q259N vectors. A) The data shows the representative images of the ERG wave forms in AAV2-Q259N injected eyes in comparison to wild type vector or mock injected rd12 mice at 32 weeks after ocular gene transfer. A rescue in physiological vision is represented by the regain in qualitative wave form. B) Dot plot for '*a wave'* and '*b wave'* plotted against the mean amplitude obtained at 3.1 log cd sec/m^2 shows significant rescue in '*a wave'* form (left graph) and '*b wave'* (right graph) in the mutant vector injected group (n=4 eyes). Values represented are mean ±SD. *p<0.05, ** p<0.01.

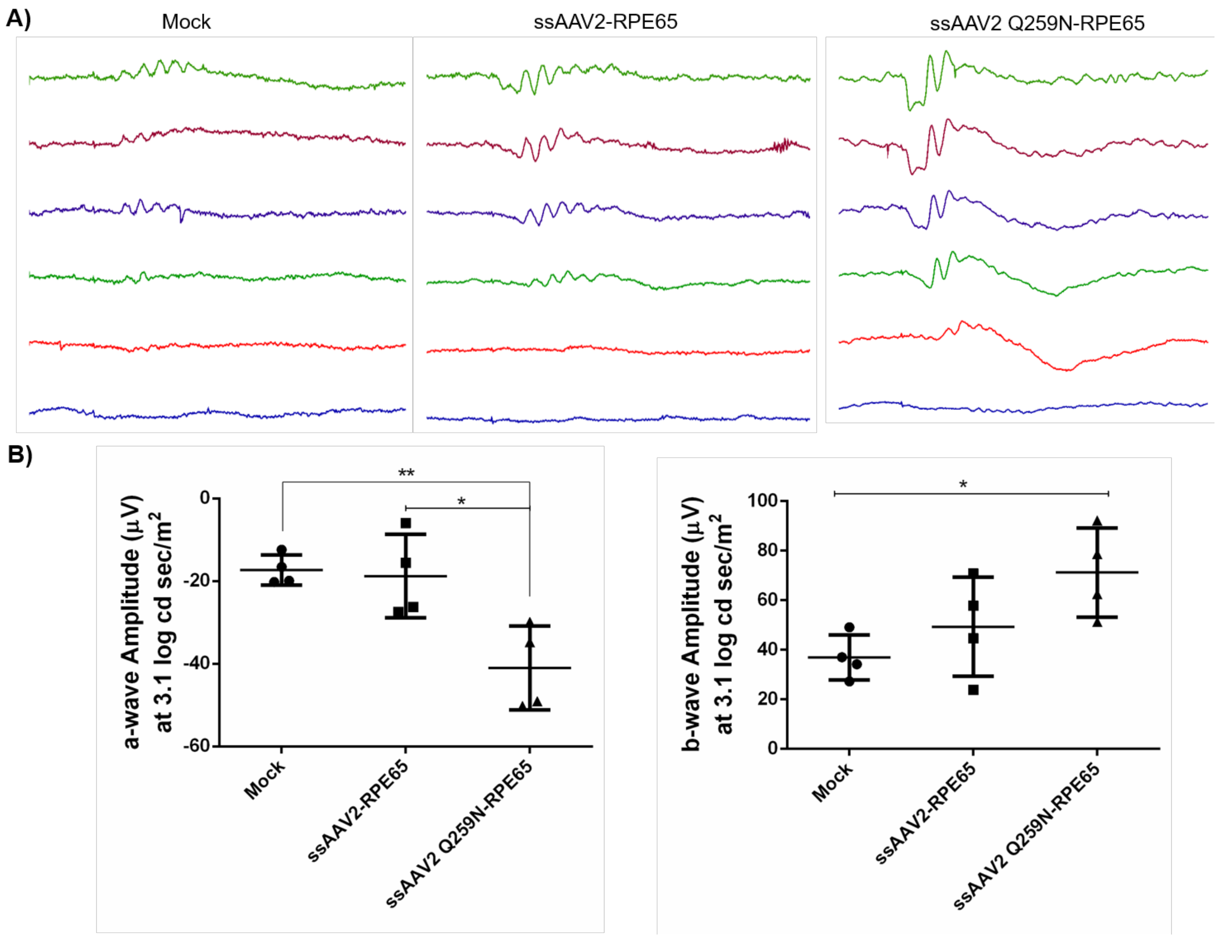

References

1. Powell, L. D., Inhibition of N-linked glycosylation. *Current Protocol in Immunol.* **2001,** *Chapter 8*, Unit 8.14.
2. Mueller, W. H.; Kleefeld, D.; Khattab, B.; Meissner, J. D.; Scheibe, R. J., Effects of retinoic acid on N-glycosylation and mRNA stability of the liver/bone/kidney alkaline phosphatase in neuronal cells. *J. Cell. physiol.* **2000,** *182* (1), 50-61.
3. Huang, J.; Byrd, J. C.; Yoon, W. H.; Kim, Y. S., Effect of benzyl-alpha-GalNAc, an inhibitor of mucin glycosylation, on cancer-associated antigens in human colon cancer cells. *Oncol. Res.* **1992,** *4* (11-12), 507-515.
4. Konrad, R. J.; Zhang, F.; Hale, J. E.; Knierman, M. D.; Becker, G. W.; Kudlow, J. E., Alloxane is an inhibitor of the enzyme O-linked N-acetylglucosamine transferase. *Biochem. Biophys. Res. Commun.* **2002,** *293* (1), 207-212.
5. Sacks, P. G.; Amos, B.; Lotan, R., Enhancement of glycosylation of cellular glycoconjugates in the squamous carcinoma cell line MDA886Ln by beta-all-trans retinoic acid. *Glycoconj. J.* **1996,** *13* (5), 791-796.
